# Chaperone-like protein DAY plays critical roles in photomorphogenesis

**DOI:** 10.1038/s41467-021-24446-5

**Published:** 2021-07-07

**Authors:** Ho-Seok Lee, Ilyeong Choi, Young Jeon, Hee-Kyung Ahn, Huikyong Cho, JiWoo Kim, Jae-Hee Kim, Jung-Min Lee, SungHee Lee, Julian Bünting, Dong Hye Seo, Tak Lee, Du-Hwa Lee, Insuk Lee, Man-Ho Oh, Tae-Wuk Kim, Youssef Belkhadir, Hyun-Sook Pai

**Affiliations:** 1 Gregor Mendel Institute (GMI), Austrian Academy of Sciences, Vienna BioCenter (VBC), Vienna, Austria; 2 Department of Systems biology, Yonsei University, Seoul, South Korea; 3 The Sainsbury Laboratory, University of East Anglia, Norwich, UK; 4 BPMP, University of Montpellier, CNRS, INRAE, Montpellier SupAgro, Montpellier, France; 5 Sainsbury Laboratory, University of Cambridge, Cambridge, UK; 6 Department of Biotechnology, College of Life Sciences and Biotechnology, Yonsei University, Seoul, South Korea; 7 Plant Developmental Genetics, Department of Biological Science, College of Biological Sciences and Biotechnology, Chungnam National University, Daejeon, South Korea; 8 Department of Life Science, Hanyang University, Seoul, South Korea

**Keywords:** Light responses, Plant morphogenesis

## Abstract

Photomorphogenesis, light-mediated development, is an essential feature of all terrestrial plants. While chloroplast development and brassinosteroid (BR) signaling are known players in photomorphogenesis, proteins that regulate both pathways have yet to be identified. Here we report that *DE-ETIOLATION IN THE DARK* *AND* *YELLOWING IN THE LIGHT (DAY)*, a membrane protein containing DnaJ-like domain, plays a dual-role in photomorphogenesis by stabilizing the BR receptor, BRI1, as well as a key enzyme in chlorophyll biosynthesis, POR. DAY localizes to both the endomembrane and chloroplasts via its first transmembrane domain and chloroplast transit peptide, respectively, and interacts with BRI1 and POR in their respective subcellular compartments. Using genetic analysis, we show that DAY acts independently on BR signaling and chlorophyll biogenesis. Collectively, this work uncovers DAY as a factor that simultaneously regulates BR signaling and chloroplast development, revealing a key regulator of photomorphogenesis that acts across cell compartments.

## Abstract (web summary)

Chloroplast development and brassinosteroid (BR) signaling both regulate plant photomorphogenesis. Here the authors show that DAY, a DnaJ-like domain-containing membrane protein, regulates both processes by binding and stabilizing both BRI1, the BR receptor, and POR, a key enzyme in chlorophyll biosynthesis.

## Introduction

After germination, seedlings must activate developmental programs appropriate to their current environment to optimize photosynthesis. When germinated in the dark, seedlings undergo skotomorphogenesis. In dicots, this results in etiolated morphologies including elongated hypocotyls, tightly closed cotyledons, and undeveloped chloroplasts. In contrast, seedlings germinated in the light undergo photomorphogenesis, resulting in short hypocotyls, open and expanded cotyledons, and the development of chloroplasts from their precursor organelles, etioplasts^1–3^. Light-dependent switching to photomorphogenic, rather than skotomorphogenic, development is critical for plant survival. The transition to photomorphogenesis depends on signaling through the brassinosteroid (BR), gibberellin (GA), and auxin hormones^1,2,4,5^.

Mutants impacting BR-biosynthesis and BR-signaling are constitutively photomorphogenic, suggesting that BR serves as a developmental bridge between skotomorphogenesis and photomorphogenesis^6–8^. BRASSINOSTEROID INSENSITIVE 1 (BRI1) is a BR receptor protein that constitutively cycles between the plasma membrane and the trans-Golgi network/early endosomal system (TGN/EE)^9^. BR signaling initiates at the plasma membrane, while the TGN/EE recycles BRI1 to the plasma membrane or mediates receptor sorting to late endosomal compartments for vacuolar delivery^10–15^. BRI1 regulates two major transcription factors, BRI1-EMS-SUPPRESSOR 1 (BES1) and BRASSINAZOLE-RESISTANT 1 (BZR1), by modulating their phosphorylation status^16,17^. In the absence of BR, BRASSINOSTEROID-INSENSITIVE 2 (BIN2) phosphorylates and inhibits BES1 and BZR1^18^. Together with PHYTOCHROME INTERACTING FACTORS (PIFs), BZR1 negatively regulates genes that are positive targets of light-signaling transcription factors^19–21^. In addition, BZR1 induces the expression of genes encoding negative regulators of photomorphogenesis, including CONSTITUTIVE PHOTOMORPHOGENIC 1 (COP1) and SUPPRESSOR OF *phyA-105* 1 (SPA1), but represses genes encoding positive light-signaling components, such as PHYTOCHROME B (PhyB) and PHOTOTROPIN 1^19,22,23^. Collectively, BR antagonistically regulates photomorphogenesis and is a major positive regulator of skotomorphogenesis.

Illumination of dark-grown seedlings triggers chlorophyll biosynthesis, and the subsequent conversion of etioplasts into photosynthetically active chloroplasts, leading to cotyledon greening^3,24,25^. The first step, triggering chlorophyll biosynthesis upon light exposure, involves the conversion of protochlorophyllide (Pchlide) to chlorophyllide through two enzymes: Pchlide oxidoreductase (POR), which functions in the light, and Pchlide oxidoreductase (DPOR), which functions in the dark. POR and DPOR catalyze conversion of Pchlide to chlorophyllide through the *trans* addition of hydrogen across the C17–C18 double bond of the D-ring of Pchlide. While both enzymes are found across oxygen-generating photosynthetic organisms, including some plants, bacteria, and algae^3,25^, flowering plants (angiosperms) have only the POR gene. Since POR-mediated catalysis requires light, chlorophyll biosynthesis arrests at Pchlide in dark-grown angiosperm seedlings^3,25^. POR accumulates in dark-grown seedlings together with Pchlide, NADPH, lipids, and carotenoids in a structure called the prolamellar body (PLB) within etioplasts. Illumination triggers degradation of POR and breakdown of PLBs, in parallel with chlorophyll synthesis and chloroplast development^3,25^. While these processes are critical to productive plant growth, a versatile factor capable of regulating the collective processes that comprise the switch to photomorphogenesis remains unknown.

Previously, we reported that CELL GROWTH DEFECT FACTOR 1 (CDF1)/CHAPERONE-LIKE PROTEIN OF POR 1 (CPP1) promotes chlorophyll biosynthesis and chloroplast biogenesis by enhancing POR stability in *Nicotiana benthamiana (N. benthamiana)* and *Arabidopsis thaliana*^26^. CPP1 deficiency impairs PLB formation in etioplasts of dark-grown plants, thus inhibiting chloroplast biogenesis upon illumination^26,27^. Though CPP1 knockdown plants have only 10% of the *CPP1* transcripts present in wild type, they eventually showed some greening upon illumination, especially in low-light conditions^26^. We therefore predicted that other factors function together with CPP1 to stabilize POR after the transition from dark to light. The *Arabidopsis* genome encodes two homologs of CPP1: CDF-RELATED GENE RESPONSIVE TO SENESCENCE (CRS) and At3g51140^28,29^. *Arabidopsis* loss-of-function *crs* mutants exhibit no visible phenotypes^29^. Thus, we focused on At3g51140, which has a DnaJ-like (JL) domain characteristic for DnaJ/Hsp40-type cochaperones predicted to have holdase chaperone activity^26,30,31^.

We observed that At3g51140 knockdown in *Arabidopsis* results in de-etiolation in the dark and leaf yellowing in the light, and thus named it *DE-ETIOLATION IN THE DARK AND YELLOWING IN THE LIGHT* (DAY). DAY is localized to chloroplasts and the endomembrane system, and this chaperone-like protein plays a dual role in photomorphogenesis. DAY functions with CPP1 in chloroplasts to promote chlorophyll biosynthesis by stabilizing POR. In the endomembrane system, DAY enhances BR signaling by interacting with and stabilizing BRI1. Our data thus point to DAY as a central, multifunctional enhancer that operates in multiple subcellular compartments and across light conditions to regulate photomorphogenic development in plants.

## Results

### DAY promotes photomorphogenesis

Previously, we analyzed the genotype of the T-DNA tagging mutant seeds of *DAY* (SALK_068109). All of the seeds examined were wild-type; there was neither *day/day* homozygous nor *DAY/day* heterozygous mutants. Therefore, we generated CRISPR/Cas9-day mutant lines. Two independent mutant lines (#1 and #2) have a large deletion in exon 2 of the *DAY* gene, 19 and 13 bp, respectively (Supplementary Fig. 1a). These mutant plants produced small siliques containing reduced numbers of seeds, compared with the WT (Supplementary Fig. 1b). Progeny analysis of CRISPR/Cas9-day lines #1 and #2 showed non-Mendelian inheritance. The genotype of the progeny was either *DAY/DAY* or *DAY/day*, but *day/day* plants have not been found, and some *DAY/day* plants showed photo-bleaching and died prematurely (Supplementary Fig. 1c–e). The lack of *day/day* genotype among progeny is consistent with the analysis of T-DNA tagging mutant lines. These results suggest that the *DAY* gene is essential for seed/embryo development and also the post-embryonic growth of *Arabidopsis* plants.

**Fig. 1:**
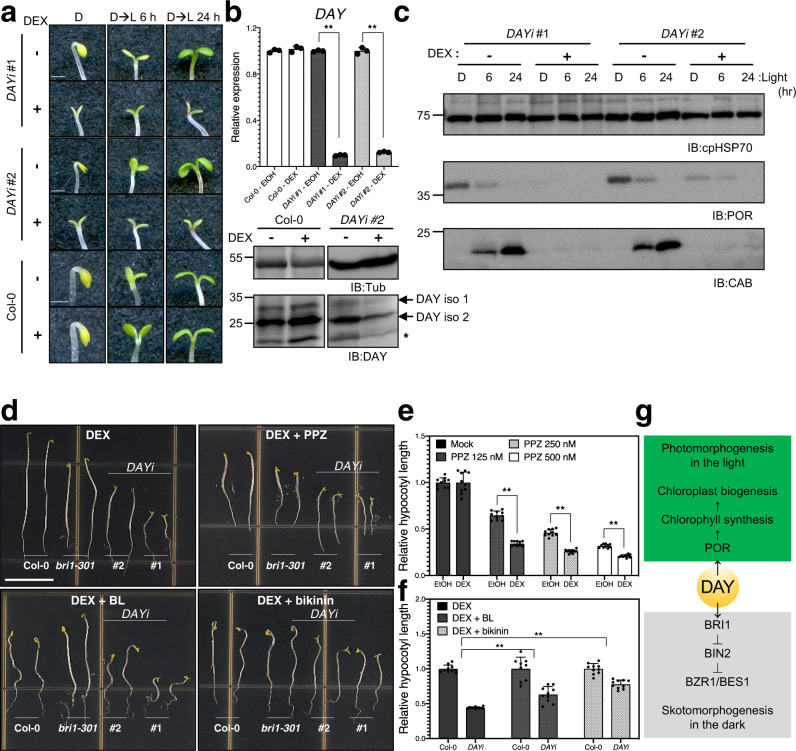
*DAY* promotes cotyledon development in the light and BR signaling in the dark. **a** Cotyledon opening responses of dexamethasone (DEX)-inducible *DAY* RNAi (*DAYi)* transgenic lines #1 and #2. Representative images of cotyledons from seedlings grown in the dark for 5 days in the presence (+) or absence (−) of 1 µM DEX and then exposed to light (D → L) for either 6 or 24 h. Scale bar, 0.1 cm. **b**, *DAY* mRNA and protein levels are significantly decreased in *DAYi* plants upon DEX treatment. Seedlings were grown in the light for 7 days in 1/2 MS medium containing ethanol (EtOH) or 1 µM DEX. RT-qPCR was used to determine *DAY* transcript levels in the knockdown plants (*upper*). Transcript levels of *DAY* were normalized relative to *PP2A A3* mRNA. Error bars indicate SD from three technical replicates. Statistical significance was determined by unpaired, two-tailed Student’s *t*-test. **P ≤ 0.01. Immunoblotting with anti-DAY antibody showed reduced DAY protein levels after DEX treatment (*lower*). The asterisk indicates a minor isoform of DAY that is consistently detected. DAY iso 1 and DAY iso 2 refer to major isoforms of DAY. **c** *DAY* promotes the accumulation of POR in the dark and CHLOROPHYLL *A/B* BINDING (CAB) proteins during the dark-to-light transition. Immunoblot analyses of seedlings (+) or (−) 1 µM DEX were exposed to light (D → L) for either 6 or 24 h. POR protein levels are very high in the dark in etioplasts, but significantly reduced upon illumination by protein degradation. Anti-POR, anti-CAB, and anti-cpHSP70 antibodies were used to analyze lysates from the two independent transgenic lines indicated on the top. **d** Reduction of *DAY* dosage phenocopies a BR signaling mutant. Morphology of 5-day-old dark-grown seedlings of wild-type, *bri1-301*, *DAYi* #1 and #2 lines grown on medium containing 1 µM DEX in the absence or presence of 250 nM propiconazole (PPZ), 50 nM brassinolide (BL), or 30 µM bikinin is shown. Scale bars for all pictures, 1 cm. **e**, **f** Hypocotyl length of 5-day-old dark-grown *DAYi* seedlings in the absence (EtOH) or presence of 1 µM DEX at increasing PPZ concentrations (**e**). Length of 5-day-old dark-grown seedlings on medium containing 1 µM DEX in the absence or presence of 50 nM brassinolide (BL) or 30 µM bikinin (**f**). Hypocotyl lengths are expressed relative to those of mock-treated samples. Values are means ± SE (*n* = 10 plants for each treatment). Statistical significance was determined by unpaired, two-tailed Student’s *t*-test. **P ≤ 0.01. **g** A model for DAY interplay with BRI1 and POR in the dark and light, respectively.

Since we were unable to obtain viable homozygote *day* mutants, we used dexamethasone (DEX)-inducible *DAY* RNAi (*DAYi*) to determine the in vivo effects of DAY deficiency in *Arabidopsis* (Fig. 1a–g). Quantitative reverse transcription PCR (RT-qPCR) and immunoblotting showed reduced *DAY* transcripts and proteins upon DEX-treatment (Fig. 1b). *DAYi* plants were grown in soil and sprayed with either ethanol (EtOH) or 1 μM DEX. After spraying plants with DEX, both vegetative- and reproductive-stage plants showed growth retardation and leaf yellowing (Supplementary Fig. 1f–h). Reproductive-stage plants treated with DEX exhibited abnormal silique development, similar to BR biosynthesis (*det2-1*) and receptor (*bri1-301*) mutants (Supplementary Fig. 1h). We obtained similar results of growth retardation and leaf yellowing using virus-induced gene silencing (VIGS) of *Arabidopsis* *DAY* (Supplementary Fig. 2). When grown on a medium containing 1 μM DEX in the dark, *DAYi* seedlings showed a de-etiolation phenotype, including short hypocotyls and open cotyledons, and were hypersensitive to the BR biosynthesis inhibitor propiconazole (PPZ) (Fig. 1a, d, and e). Furthermore, the shortened hypocotyl de-etiolation phenotype of *DAYi* plants was partially rescued by exogenous treatment with Brassinolide (BL) or bikinin, an inhibitor of *Arabidopsis* BIN2 kinases, suggesting DAY promotes BR signaling (Fig. 1d, f and Supplementary Fig. 3). In addition, DAY knockdown plants exhibited reduced cotyledon greening upon transfer from dark to light conditions (Fig. 1a). This impairment in de-etiolation was associated with a reduced POR abundance in the dark and a failure to accumulate CHLOROPHYLL A/B BINDING protein (CAB) during the dark-to-light transition (Fig. 1c). Neither *crs* nor BR signaling mutants exhibited POR instability or impaired cotyledon greening in the dark-to-light transition (Supplementary Figs. 4 and 5). These results indicate that DAY is uniquely and specifically involved in two distinct photomorphogenesis processes (Fig. 1g).

### Multiple subcellular localization of DAY

**Fig. 2:**
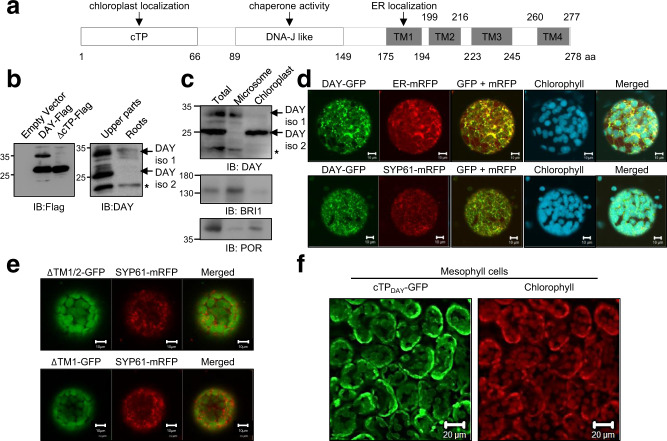
Localization of DAY in chloroplasts and the endomembrane system depends on the cTP and TM1, respectively. **a** Schematic representation of DAY with its chloroplast transit peptide (cTP), DnaJ-like domain (JL) and four transmembrane domains (TM1–4). The amino acid (aa) positions that define the domains are indicated at the bottom. **b** DAY exists in two major isoforms in vivo. *N. benthamiana* leaves expressing either full-length FLAG-tagged DAY (DAY-Flag) or a variant lacking the chloroplast transit peptide (∆cTP-Flag) were subjected to immunoblotting (IB) analysis using anti-Flag antibodies (left panel). Immunoblot analysis of aerial and root tissues from wildtype *Arabidopsis* seedlings using anti-DAY antibodies (right panel). DAY iso 1 and DAY iso 2 refer to major isoforms of DAY. The asterisk indicates a minor isoform of DAY that is consistently detected. **c** Subcellular fractionation. WT seedling extracts were fractionated and subjected to immunoblotting with an anti-DAY antibody. BRI1 and POR were detected as the markers for microsomal fractions and chloroplasts, respectively. **d** DAY localizes to chloroplasts, ER, and TGN/EE. Co-localization of DAY with ER-mRFP (upper panel), SYP61-mRFP (lower panel), and chlorophyll support is shown. mRFP and DAY-GFP fusion proteins were transiently co-expressed in *N. benthamiana* leaves by agroinfiltration, and protoplasts isolated from infiltrated leaves were imaged using confocal laser scanning microscopy (CLSM). SYP61 (SYNTAXIN OF PLANTS 61) was used as a TGN/EE marker. Scale bar, 10 µm. **e** TM1 is responsible for ER and TGN/EE localization of DAY. GFP fusions to a DAY deletion series were co-expressed with SYP61-mRFP in *N. benthamiana* leaves via agroinfiltration and protoplasts were observed by CLSM. Scale bar, 10 µm. **f** The DAY cTP is sufficient for chloroplast targeting. cTP_DAY_-GFP fusion proteins were expressed in *N. benthamiana* leaves via agroinfiltration and mesophyll cells were imaged using CLSM. Scale bar, 20 µm.

Chlorophyll biogenesis takes place in the chloroplast while the BR signal transduction cascade occurs between the cell surface and nucleus^3,9^, raising the question of which subcellular compartment(s) DAY functions in. DAY is predicted to have a chloroplast transit peptide (cTP) that has to be cleaved during translocation from cytosol to chloroplast, a JL domain for holdase activity, and 4 transmembrane domains (Fig. 2a). We transiently expressed Flag-tagged DAY (DAY-Flag) and a deletion mutant lacking the cTP (∆cTP-Flag) in *N. benthamiana* leaves. We detected signals above 35 kDa (DAY iso 1) and signals above 25 kDa (DAY iso 2), indicating that DAY exists in two isoforms (Fig. 2b). To confirm that endogenous DAY is present in two isoforms, we generated anti-DAY antibodies and performed immunoblotting of aerial tissues and roots from *Arabidopsis* seedlings (Fig. 2b). In both tissues, we detected DAY iso 1, but DAY iso 2 was only detected in aerial tissues. To determine the subcellular localization of two isoforms of DAY, we performed subcellular fractionation using WT seedling extracts, followed by immunoblotting with anti-DAY antibody (Fig. 2c). Using immunoblotting with anti-BRI1 and anti-POR antibodies, BRI1 and POR were detected as the markers for microsomal fractions and chloroplasts, respectively. The microsomal fractions contained the larger DAY protein (DAY iso 1), while chloroplast fractions contained the processed smaller form of DAY (DAY iso 2).

Next, we examined the subcellular localization of DAY using fluorescent fusion proteins expressed in *N. benthamiana* leaves. We used confocal microscopy to compare the localization of Arabidopsis DAY fused to mRFP (DAY-mRFP) with markers for the endoplasmic reticulum (ER-GFP), the TGN/EE (TGN/EE-GFP), and chloroplasts (chlorophyll auto-fluorescence) in leaf epidermal cells. We detected DAY-mRFP at the plasma membrane and also found it colocalized with the chloroplasts, TGN/EE-GFP, and ER-GFP (Supplementary Fig. 6a). We confirmed this localization in *N. benthamiana* protoplasts using fluorescent protein combinations (Fig. 2d and Supplementary Fig. 6b). We also examined the localization of DAY-GFP in 35S::DAY-GFP transgenic *Arabidopsis* plants. Cotyledon cells from dark-grown plants and root cells from light-grown plants had DAY-GFP signals at the plasma membrane, in intracellular mesh-like structures, and as punctate spots within the cytoplasm (Supplementary Fig. 6c).

To confirm the broad subcellular distribution of DAY, we performed RT-qPCR for *DAY* and 4 other genes encoding chloroplast-localized proteins in chloroplast-rich aerial tissues and roots from light-grown WT seedlings. We found that levels of *DAY* mRNA in roots were significantly higher than the other chloroplast-localized proteins, consistent with DAY expression in tissues lacking chloroplasts (Fig. 2b and Supplementary Fig. 6d). Collectively, these results demonstrate that DAY is localized to both chloroplasts and the endomembrane system.

### Different domains of DAY control its subcellular location

To identify the domains controlling DAY localization, we expressed deletion mutants in *N. benthamiana* protoplasts and mesophyll cells and compared localization with markers for chloroplasts (chlorophyll), the ER (ER-mRFP), and the TGN/EE (SYP61-mRFP) (Fig. 2e and Supplementary Figs. 7 and 8). Deletion of the first transmembrane domain alone or in combination with the second transmembrane domain resulted in DAY-GFP accumulation in round structures resembling chloroplasts and prevented TGN/EE localization (Fig. 2e). Deletion of the cTP alone or in combination with the 3rd and 4th transmembrane domains individually did not affect the localization of DAY to the endomembrane system (Supplementary Figs. 7 and 8). These results indicated that the first transmembrane domain controls DAY localization to the TGN/EE. To determine if the cTP is sufficient to localize proteins to the chloroplast, we fused the cTP from DAY to GFP. cTP-GFP colocalized with chlorophyll when expressed in *N. benthamiana* mesophyll cells (Fig. 2f). Taken together, these results suggest that DAY protein exists in two forms, and the first transmembrane domain and the cTP region are critical for targeting DAY protein to the endomembrane system and chloroplasts, respectively.

### DAY exhibits chaperone activity for POR

**Fig. 3:**
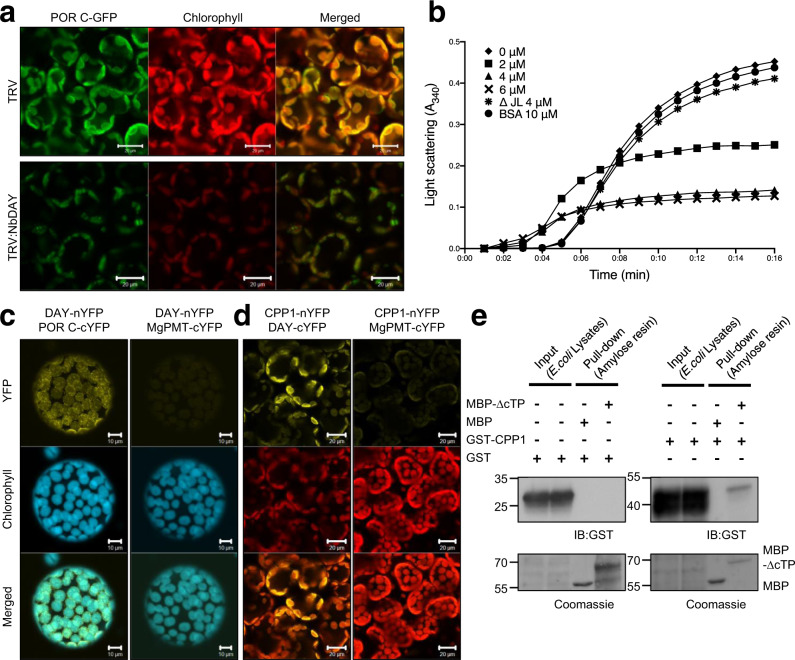
Together with CPP1, DAY regulates the stability of protochlorophyllide oxidoreductase (POR) through its holdase chaperone activity. **a** Reduction of DAY causes POR instability in *N. benthamiana*. Virus-induced gene silencing (VIGS) of *N. benthamiana DAY* (*NbDAY*) was performed. Then the VIGS plants (TRV:NbDAY) were agroinfiltrated with the POR C-GFP construct, and leaf mesophyll cells were examined by CLSM. POR C is a major POR isoform in light-grown Arabidopsis. *NbDAY* silencing caused a significant reduction in chlorophyll autofluorescence and POR C-GFP fluorescence. Scale bar, 20 µm. **b** DAY has holdase activity. Absorbance at 340 nm (*A*_340_) was measured at 1 min intervals to quantify turbidity. Thermal aggregation of malate dehydrogenase (2 µM) was measured at 43 °C over 16 min with 0–6 µM of MBP-∆cTP DAY or, as controls, 4 µM MBP-∆cTP/∆JL DAY (del JL) and 10 µM BSA. ∆cTP, deletion of cTP; ∆JL, deletion of the DnaJ- like domain (JL). **c**, **d** DAY interacts with POR C and CPP1 in chloroplasts. BiFC assays between DAY and POR C (**c**) or CPP1 (**d**). YFP signals were examined by CLSM in protoplasts after agroinfiltration. MAGNESIUM-PROTOPORPHYRIN IX METHYL TRANSFERASE (MgPMT), which is involved in chlorophyll biosynthesis, was used as a negative control. Scale bar, 10 µm (**c**) or 20 µm (**d**). **e** DAY directly interacts with CPP1 in vitro. Lysates of *E. coli* cells expressing GST or GST-CPP1 were incubated with MBP or MBP-∆cTP DAY (MBP-∆cTP) immobilized on amylose agarose resins. The resulting eluates were analyzed by immunoblotting using anti-GST antibody. The amount of MBP and MBP-∆cTP used in the pull-down assay were visualized by Coomassie staining.

Given that DAY knockdown results in reduced POR levels and prevents chloroplast development (Fig. 1a, c), we tested whether DAY controls POR stability. VIGS of *DAY* in *N. benthamiana* resulted in leaf yellowing and growth retardation with spontaneous cell death, accompanied by reduced POR protein levels (Supplementary Fig. 9). We used CLSM to examine chloroplasts and GFP-fused POR C, a major POR isoform after illumination in *Arabidopsis*^25^, in *DAY* VIGS *N. benthamiana* plants. *DAY* knockdown caused a significant reduction in chlorophyll autofluorescence and POR C-GFP fluorescence, suggesting that DAY protein is critical for maintaining chloroplast integrity and POR C accumulation (Fig. 3a). To determine if DAY has chaperone activity, we assessed its ability to prevent heat-induced denaturation and aggregation of proteins in vitro. We found that maltose-binding protein (MBP)-fused ∆cTP DAY (MBP-∆cTP) suppressed thermal aggregation of malate dehydrogenase (MDH) in a concentration-dependent manner (Fig. 3b). The addition of DAY lacking the JL domain (∆JL) failed to prevent MDH aggregation, indicating that the JL domain is required for chaperone activity (Fig. 3b).

We next tested for direct interaction between DAY and either POR or CPP1 using bimolecular fluorescence complementation (BiFC), in which the N- and C-terminal portions of yellow fluorescent protein (nYFP and cYFP) are separately fused to the candidate interacting proteins. We expressed DAY and POR as nYFP and cYFP fusion proteins in *N. benthamiana* leaves and visualized protoplasts by CLSM. We observed strong YFP fluorescence within chloroplasts, indicating an interaction between DAY and POR (Fig. 3c). As a negative control, we performed BiFC with DAY and MAGNESIUM-PROTOPORPHYRIN IX METHYL TRANSFERASE (MgPMT), which also localizes to the chloroplast. We also observed a positive signal for BiFC using between CPP1 and DAY, but not CPP1 and MgPMT (Fig. 3d). In vitro pull-down assays further confirmed a direct interaction between immobilized MBP- ▵cTP and glutathione S-transferase (GST)-fused CPP1 (GST-CPP1), but not with GST. GST-CPP1 specifically bound MBP-▵cTP but not MBP alone (Fig. 3e). Given that DAY has a JL domain similar to those found in DnaJ/Hsp40 cochaperones that function as partners of the highly conserved Hsp70^30,31^, we performed BiFC to test whether DAY interacts with chloroplast Hsp70 (cpHsp70) or cytosolic Hsp70 (Hsp70) (Supplementary Fig. 10). As BiFC controls, co-expression of Hsp70 and Hsp101 (cytosolic), and cpHsp70 and Hsc90 (chloroplast) resulted in yellow fluorescence. The lack of fluorescence despite the abundant protein expression suggested that DAY interacts with neither cpHsp70 nor Hsp70. This is likely caused by the fact that DAY lacks the HPD (His-Pro-Asp) motif^30,31^ that is critical for interaction with Hsp70^26^. Collectively, these data demonstrate that DAY in the chloroplast stabilizes POR C with its holdase chaperone activity and also interacts with CPP1.

### DAY alters BRI1 stability and enhances BR signaling

**Fig. 4:**
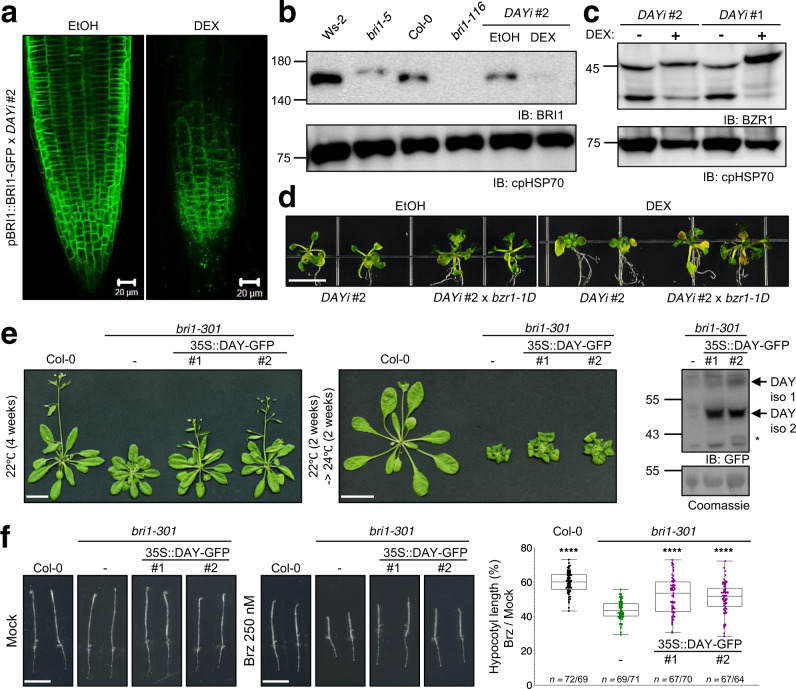
DAY regulates BRI1 stability. **a** DAY deficiency triggers BRI1 instability. *DAY* silencing causes aggregation of BRI1-GFP in *Arabidopsis* roots. The pBRI1::BRI1-GFP × *DAYi* seedlings were grown in medium containing ethanol or 1 µM DEX in the light for 9 days, and their roots were observed by CLSM. Scale bar, 20 µm. **b** *DAY* silencing decreases BRI1 protein levels. 9-day-old seedlings of WT (Ws-2 and Col-0), *bri1* mutants (*bri1–5* and *bri1-116*), and *DAYi* plants grown in the presence of ethanol or 1 μM DEX were subjected to immunoblotting with anti-BRI1 and anti-cpHSP70 antibodies. **c** *DAY* silencing increases phosphorylated BZR1. 9-day-old *DAYi* seedlings grown in the presence of ethanol or 1 μM DEX were subjected to immunoblotting using anti-BZR1 and anti-cpHSP70 antibodies. **d**, The dominant mutation *bzr1-1D* rescues the short petiole phenotype, but not chloroplast defect, of *DAY*-silenced seedlings. Seedlings from *DAYi* and *DAYi* x *bzr1-1D* lines were grown on 1/2 MS medium for 5 days at 22 °C and then transferred to medium containing ethanol or 1 µM DEX at 28 °C for 7 days. Scale bar, 1 cm. **e**, **f**, Overexpression of DAY partially rescues a BR signaling mutant. Plants were grown in soil for 4 weeks at 22 °C (**e**, *left panel*) or 2 weeks at 22 °C and then transferred to 24 °C for 2 weeks (**e**, *middle panel*). Expression of DAY-GFP protein in *bri1-301* mutant was shown by immunoblotting with anti-GFP antibody (**e**, *right panel*). Morphology of 5-day-old dark-grown seedlings of wild-type, *bri1-301*, 35 S::DAY-GFP / *bri1-301* #1 and #2 transgenic lines on medium in the absence (**f**, *left panel*) or presence of 250 nM brassinazole (Brz) (**f**, *middle panel*). Hypocotyl lengths are quantified relative to those of mock-treated samples (**f**, *right panel*). Genotypes are indicated. Dots represent individual ratio values (%) between mock-treated and Brz-treated independent seedlings. *n* denotes numbers of biologically independent hypocotyls, Brz- *(left number*), and mock-treated (*right number*). Statistical significance was determined by one-way ANOVA, Dunnett’s multiple comparisons test ****P < 0.0001. Scale bars: 3 cm (**e**) or 1 cm (**f**).

Since the DAY chaperone is in the same compartment as BRI1, we tested whether it affects BRI1 stability. We monitored BRI1-GFP fluorescence in transgenic *Arabidopsis* seedlings (pBRI1::BRI1-GFP × *DAYi*) in response to *DAY* knockdown. BRI1-GFP aggregates accumulate in *DAY* knockdown *Arabidopsis* roots (Fig. 4a). To determine whether general vesicular transport is affected in *DAY* knockdown plants, we followed endocytosis by labeling roots with the endocytic tracer FM4-64 and marked the TGN/EE using the vacuolar H^+^ ATPase A1 subunit (VHA-a1-RFP). *DAY* knockdown did not affect endocytosis or TGN/EE distribution in roots (Supplementary Fig. 11).

To determine whether DAY increases BRI1 kinase activity, we performed peptide kinase assays using the synthetic peptide BR13^32^. We isolated a Flag-tagged version of the BRI1 cytosolic domain (Flag-BRI1:CD) from *E. coli* before and after induction of kinase expression^33^ and monitored kinase activity in the presence of MBP alone or fused to DAY (MBP-DAY). BRI1 kinase activity on the BR13 peptide was similar in the presence of MBP or MBP-DAY, indicating that DAY does not enhance BRI1 kinase activity (Supplementary Fig. 12). Although the addition of DAY to the in vitro kinase reaction did not affect BRI1 activity, immunoblotting of *DAY* knockdown plants grown in the light revealed decreased BRI1 levels (Fig. 4b and Supplementary Fig. 13) and increased BZR1 phosphorylation (i.e., inactive BZR1; Fig. 4c). These results indicate that DAY promotes BR signaling.

We next genetically tested whether DAY positively regulates BR signaling. We knocked down *DAY* with RNAi in *brassinazole resistant1–1D (bzr1-1D)* plants. *bzr1-1D* mutants carry a dominant gain of function mutation in *BZR1*, resulting in a constitutive BR response^17^. Following DEX treatment, *DAYi* wild-type and *DAYi* × *bzr1-1D* plants both displayed leaf yellowing phenotypes, while only *DAYi* plants displayed extremely short petioles (Fig. 4d and Supplementary Fig. 14a), indicating that the short petiole phenotype of *DAY*-silenced seedlings resulted from impaired BR signaling. RT-qPCR experiments with *DAYi* wild-type seedlings and *DAYi*  ×  *bzr1-1D* seedlings confirmed effective DEX-induced knockdown of DAY and showed that *bzr1-1D* rescued the decrease in BR-related gene expression in *DAY*-silenced plants (Supplementary Fig. 14b). If DAY stabilizes BRI1, we predicted that DAY overexpression would enhance BR signaling. Immunoblotting of 35S::DAY-GFP showed that DAY enhances BRI1 protein accumulation in *Arabidopsis* (Supplementary Fig. 15). As BR regulates hypocotyl elongation, we measured hypocotyl length of 35S::DAY-GFP plants in the dark with or without BR biosynthesis inhibitor brassinazole (Brz). DAY overexpression seedlings showed longer hypocotyls upon Brz treatment compared with WT seedlings, indicating reduced sensitivity to Brz (Supplementary Fig. 16). Lastly, we introduced 35S::DAY-GFP to the weak *bri1* mutant allele *bri1-301*. DAY overexpression partially rescues the thermosensitive dwarfism^34^ and growth retardation phenotypes in *bri1-301* (Fig. 4e). We also measured hypocotyl length of 35S::DAY-GFP / *bri1-301* in the dark with or without Brz. DAY overexpression partially rescued Brz hypersensitivity in *bri1-301* in the dark (Fig. 4f).

Since DAY enhances BR signaling, possibly via BRI1 stabilization, we performed RNA-Seq on seedlings of *DAYi* and the *bri1* null mutant allele *bri1-116*. We compared the transcriptomes of *DAYi* and *bri1-116* seedlings grown in the dark or light. We found that the transcriptome of *DAYi* seedlings significantly overlaps with that of the *bri1-116* mutant, especially in the dark (Supplementary Figs. 17–19). Collectively, these data indicate that DAY acts as a positive regulator of BR signaling.

### DAY interacts with BRI1 in the endomembrane system

**Fig. 5:**
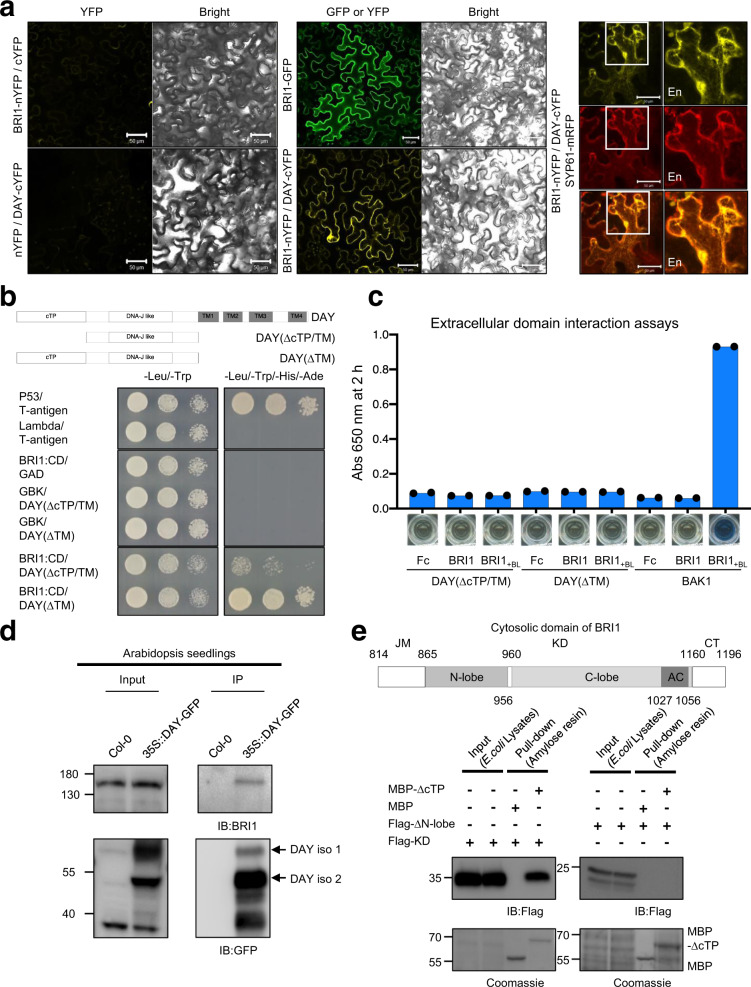
DAY directly interacts with BRI1. **a** DAY interacts with BRI1 in TGN/EE and plasma membrane. BiFC between BRI1-nYFP and DAY-cYFP results in yellow fluorescence, suggesting protein interaction in vivo (middle, lower panel). BiFC fluorescence was compared with SYP61-mRFP fluorescence, which marks the TGN/EE (right panel). *N. benthamiana* epidermal cells were observed two days after agroinfiltration. Enlarged views of the *boxed* areas are shown (*En*). Scale bar, 50 µm. **b** DAY directly interacts with the BRI1 cytosolic domain. Yeast two-hybrid assays suggest that both DAY deletion variants (∆cTP/TM and ∆TM) interact with the cytosolic domain of BRI1. The p53/T-antigen and Lambda/T-antigen combinations were used as positive and negative controls, respectively. CD, the cytosolic domain; ∆TM, deletions of all four TMs. **c** Extracellular domain interaction assays show that neither of the DAY deletion variants, ΔcTP/TM and ΔcTP, interact with the extracellular domain of BRI1. DAY variants and the extracellular domain of BRI1 are expressed as prey and bait, respectively. The prey was fused with alkaline phosphatase (AP) and the bait with the fragment crystallizable region (Fc) from the tail region of the antibody. BAK1/BRI1 with brassinolide (+BL) was used as a positive control. (*n* = 2 technical replicates) **d** Co-immunoprecipitation shows that DAY interacts with BRI1. Total *Arabidopsis* seedling proteins were immunoprecipitated with GFP-Trap Agarose, and co-immunoprecipitated proteins were detected using the anti-BRI1 antibody. Two isoforms of DAY protein, DAY iso1, and iso2, are indicated. **e** Deletion of the N-terminal lobe in the BRI1 kinase domain reduces its binding affinity for DAY. *Upper panel*; diagram of the cytosolic domain of BRI1. JM juxta-membrane domain, KD kinase domain, CT C-terminal tail, N-lobe N-terminal lobe, C-lobe C-terminal lobe, AC activation loop. Amino acid residue numbers are indicated. *Lower panel*; lysates of *E. coli* cells expressing the Flag-tagged KD of BRI1 (Flag-KD) or the Flag-tagged KD of BRI1 lacking the N-terminal lobe (Flag-∆N-lobe) were incubated with MBP or MBP-∆cTP DAY (MBP-∆cTP) immobilized on amylose agarose resins. Eluates were analyzed by immunoblotting with anti-Flag antibody or Coomassie staining.

Since DAY promoted BR signaling via BRI1, we used BiFC to test whether DAY and BRI1 interact in the endomembrane system. Co-expression of BRI1-nYFP and DAY-cYFP resulted in YFP fluorescence in the plasma membrane and at intracellular puncta (Fig. 5a). The puncta-localized BiFC interaction signal co-localized with SYP61-mRFP, pointing to the TGN/EE as sites of DAY-BRI1 interaction (Fig. 5a). To confirm this interaction and define interacting domains, we performed yeast two-hybrid (Y2H) assays, extracellular domain interaction assays (EDIA)^35^, and pull-down assays. In the Y2H assays, DAY strongly interacted with the cytosolic domain of BRI1 (BRI1:CD) in the presence of the cTP (Fig. 5b). Extracellular domain interaction assays (EDIA) revealed that DAY does not interact with the extracellular domain of BRI1 (Fig. 5c). Finally, immobilized Flag-BRI1:CD pulled down both MBP-DAY and MBP-▵cTP, but not MBP (Supplementary Fig. 20a), consistent with the Y2H results (Fig. 5b). The cTP sequence stimulated the binding between DAY and BRI1 CD in both the Y2H and the pull-down experiments. We found that MBP-DAY also interacts with cytosolic domain of BRI1-Like 1 (BRL1), a BRI1 homolog, via pull-down assays (Supplementary Fig. 20b). Collectively, these results indicate that DAY directly interacts with the cytosolic kinase domain of BRI1, but not with its extracellular domain.

To confirm the interaction between full-length BRI1 and DAY in vivo, we performed co-immunoprecipitation assays using total proteins from transgenic 35S::DAY-GFP *Arabidopsis* seedlings with WT (Col-0) as control (Fig. 5d). DAY-GFP protein was pulled-down by GFP-Trap Agarose. Then immunoblotting with anti-BRI1 antibody detected native BRI1 protein as co-immunoprecipitates, suggesting in vivo interactions between BRI1 and DAY.

To determine which cytosolic region of BRI1 interacts with DAY, we performed in vitro binding assays. The cytosolic domain of BRI1 consists of the (i) juxta-membrane domain (JM), (ii) kinase domain (KD), which contains an N-terminal lobe (N-lobe), C-terminal lobe (C-lobe), and activation loop (AC), and (iii) C-terminal tail (CT) (Fig. 5e). Immobilized Flag-BRI1 KD with either the JM or CT domain, as well as the full-length cytosolic domain of BRI1, pulled-down MBP-DAY (Supplementary Fig. 20c). To examine the region of the BRI1 KD responsible for binding DAY, we performed in vitro binding assays using purified BRI1 KD (Flag-KD) or BRI1 KD without the N-lobe (Flag-▵N-lobe) and recombinant MBP-▵cTP or MBP. Deletion of the N-lobe from the BRI1 KD significantly reduced the interaction with MBP-▵cTP, suggesting the importance of the N-lobe sequence for the interaction (Fig. 5e). Together, these results suggest that the N-lobe of BRI1 KD and the cTP of DAY may be critical for interactions between DAY and BRI1 KD.

## Discussion

In this study, we show that DAY is a chaperone-like protein that may stabilize BRI1 and POR. Through deletion analysis, we find that DAY localizes to the chloroplast through its cTP and to the endomembrane via its first transmembrane domain. DAY also has a DnaJ-like domain with a holdase activity that prevents protein aggregation in vitro. Despite the importance of the DnaJ-like domain, there is a possibility that other regions of DAY protein may also be required for its substrate-stabilizing activity in vivo. Our results demonstrate that DAY regulates photomorphogenesis through two different pathways: BR signaling and chlorophyll biogenesis. These findings uncover an unexpected link between BR signaling and chloroplast development. While some proteins have been shown to be transported to the chloroplast via the secretory pathway, this is, to our knowledge, the first evidence that such a protein functions in both subcellular compartments^36,37^.

We previously reported that CPP1 not only protects POR from photo-oxidative damage but may also contributes to anchoring POR to the thylakoid and envelope membrane^26^. However, salt extraction experiments indicated that CPP1 is not as tightly bound to the chloroplast membranes as would be expected for an intrinsic membrane protein, given that CPP1 has four transmembrane domains^27^. DAY may play a role in anchoring POR to the thylakoid and envelope membrane together with CPP1 (Fig. 3a). Indeed, BiFC showed that the majority of the interactions between CPP1 and DAY occur in the chloroplast envelope membrane (Fig. 3d). Monitoring POR protein aggregation revealed that a 2 fold-higher molar ratio of CPP1 is required to protect POR from oxidative stress^26^. POR is the most abundant protein within PLB in etioplasts^3,25^, suggesting that another factor or protein also protects POR from photo-oxidative stress upon illumination. DAY may work together synergistically with CPP1 to protect POR.

The most well-described chaperone for kinase is the Hsp90–Cdc37 (chaperone-cochaperone) pair in animals, which interacts with diverse kinases^38^. A deletion study of lymphocyte-specific protein tyrosine kinase showed that the Hsp90–Cdc37 pair binds to the N-lobe of the kinase domain^39^. Chaperone seems to bind to the intrinsically unstable N-lobe, stabilizing its substrate^38^. In some kinases, e.g., C-Jun N-terminal kinases, the N-lobe can be stabilized by a polypeptide motif within the same protein^40^. Caplan et al. ^38^ demonstrated that the stability of the N-lobe determines whether kinases interact with chaperones. Indeed, the crystal structure of the phosphorylated BRI1 kinase domain revealed that phosphorylation of Thr872 within the N-lobe of BRI1, which is essential for kinase activity^33^, hindered crystallization. They were only able to obtain crystals after mutating Thr872 to alanine^41^, indicating that phosphorylated BRI1 may be intrinsically unstable. Furthermore, DAY binds to BRI1 mainly via the N-lobe of its kinase domain (Fig. 5e). In contrast, LORELEI-LIKE GLYCOSYL PHOSPHATIDYL INOSITOL-ANCHORED PROTEIN 1 (LLG1) acts as a chaperone that delivers the receptor-like kinase FERONIA (FER) to the plasma membrane by interacting with its extracellular domain. In the *llg1-2* mutant, FER fails to reach the plasma membrane and remains trapped in the ER^42^, similar to our observation that BRI1 fails to reach the plasma membrane in *DAYi* (Fig. 4a). Collectively, we propose that the N-lobe in the BRI1 kinase domain is intrinsically unstable, and that DAY may serve as a chaperone to stabilize it.

**Fig. 6:**
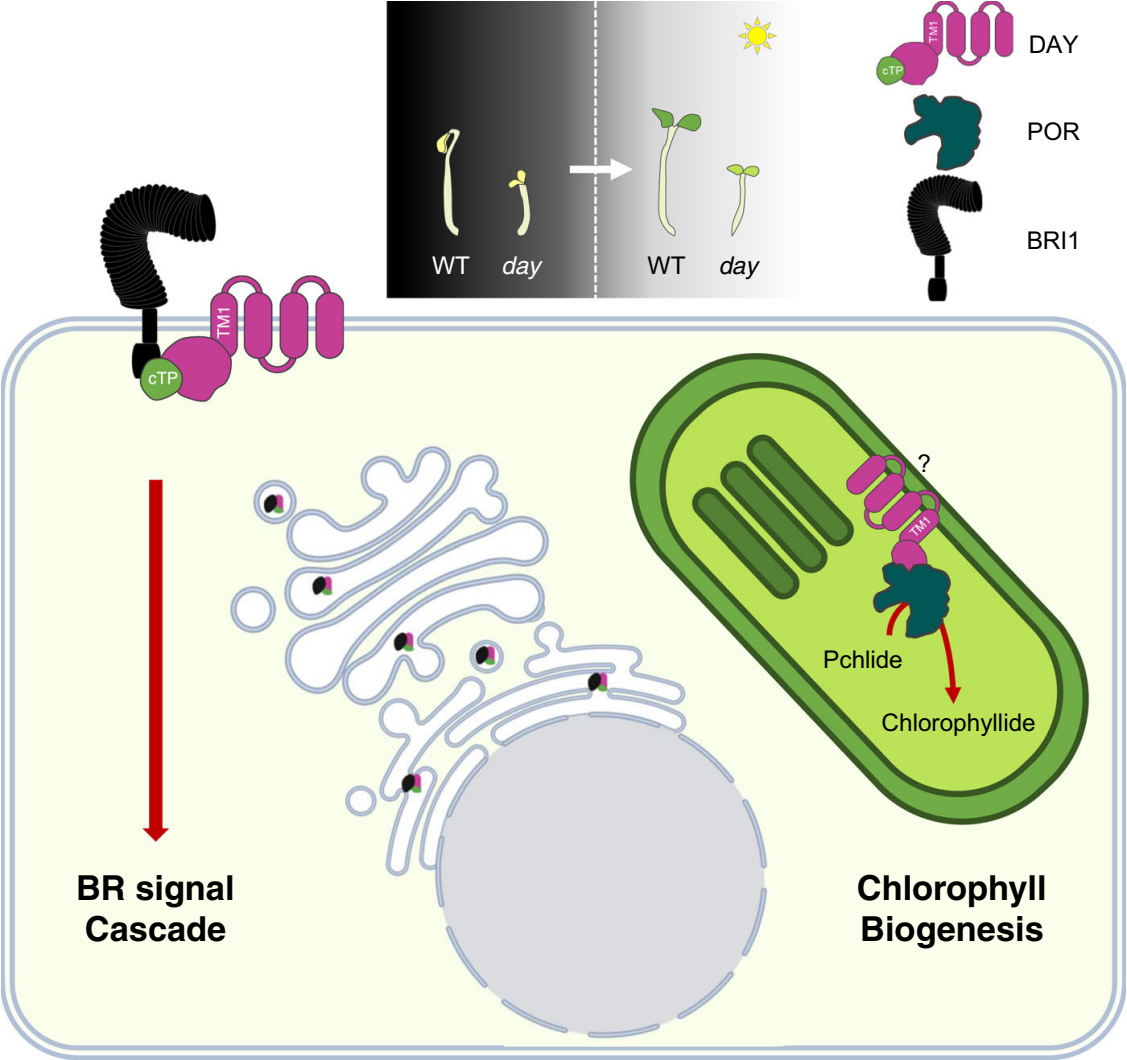
A model for stabilization of BRI1 and POR by DAY in different subcellular compartments. DAY binds to and stabilizes BRI1 and POR in the endomembrane system and chloroplast, respectively. The cTP sequence is sufficient to transfer DAY to the inner envelope membrane of chloroplast and the first transmembrane domain is required for DAY localization in ER, TGN/EE, and plasma membrane. DAY-mediated BR signaling and chlorophyll biosynthesis are essential for photomorphogenesis. The illustration of nuclear envelope-ER-Golgi is purchased from Biorender.

Looking back at the incredible stress placed on a seedling exposed to light for the first time, we favor a model in which DAY mediates photomorphogenic development directly in both the endomembrane system and chloroplasts (Figs. 1g and 6). In this way, a plant can initiate a rapid and harmonized response to the first exposure, and then adjust to the ambient light environment as it adapts. A similar model has been proposed for the orchestrated action of the nuclear- and chloroplast-localized protein HEMERA (HMR) during photomorphogenesis. Nuclear HMR is required for the proteolysis of PHYA, PIF1, and PIF3, while chloroplast HMR directly regulates the expression of photosynthetic genes as a transcriptional regulator^43–47^.

The transition from skotomorphogenesis to photomorphogenesis is a dramatic developmental change that entails transcriptional changes in one-third of all genes, as seen in 9216 expressed sequence tag analysis^48^. This developmental transition is critical; since dark-germinating seedlings have limited energy reserves, they must transition to photosynthetic autotrophy as quickly as possible when provided with light. Morphological and biochemical changes become obvious within 6 h of light exposure^1,2,22^ and analysis of light-regulated genes found that more than 26 cellular pathways are affected^48^. Indeed, the mechanism we have uncovered, where a single protein simultaneously regulates multiple pathways in different subcellular compartments, would explain this fast, efficient transition occurring in the cell. Moreover, there is an obvious biological advantage to have a single protein localized to multiple compartments, since the number of genes that provide the same function in different subcellular compartments can be reduced, and the switch can be easily coordinated. For example, about 100 proteins are localized to both the mitochondria and chloroplasts, where many of them provide similar functions, such as organellar DNA replication and protein synthesis^49^. DAY may have been co-opted to regulate morphogenetic change and chloroplast development through BR signaling and chlorophyll biogenesis, respectively. It is interesting to note that a homolog of the higher-plant DAY/CDF family genes has been found in cyanobacteria^26^. The ubiquitous presence of DAY across the plant kingdom and cyanobacteria suggests an essential role for DAY in light-regulated development.

## Methods

### Plant materials and growth conditions

The *A. thaliana* Col-0 accession was used throughout this study, except for the *bri1-5* mutant which is in the Ws-2 background. *A. thaliana* and *N. benthamiana* plants were grown in a growth room at 22 °C under 16h-light/8h-dark (long day) conditions. To grow seedlings, seeds were surface-sterilized and grown on plates with 1/2 Murashige and Skoog (MS) with 1% sucrose and 0.8% phyto agar at 22 °C under long-day conditions.

### Plasmid construction

All plasmids were constructed using PCR-based conventional cloning or the Gateway system (Invitrogen^TM^;TOPO™ TA Cloning™ or GreenGate). Detailed information is listed in Supplementary Data 1.

### Silique clearing

Mature siliques were fixed overnight in ethanol:acetic acid (3:1) solution at room temperature. The next day, siliques were cleared with modified Hoyer’s solution (200% chloral hydrate and 20% glycerol in Mono Q). Siliques were mounted in solution on a slide-glass and observed with a LEICA M165 FC. Representative pictures of siliques were taken with a LEICA DFC 7000T.

### *Agrobacterium tumefaciens*-mediated transient expression

*Agrobacterium* C58C1 strains containing various expression constructs were resuspended to OD600 = 1 in MES buffer (10 mM MES, pH 7.5, 10 mM MgSO_4_, and 200 μM acetosyringone) after overnight culture. After 2–3 h incubation at room temperature, the suspension was infiltrated into *N. benthamiana* leaves. *Agrobacterium* C58C1 carrying the 35S::p19 constructs was co-infiltrated to enhance expression. Leaves were subjected to immunoblotting or analyzed by confocal microscopy 3 days after infiltration.

### Co-immunoprecipitation assays and immunoblotting

For *N. benthamiana*, samples were prepared by grinding leaves in liquid nitrogen and immediately extracted with Co-immunoprecipitation (co-IP) buffer (50 mM Tris–HCl pH 7.5, 150 mM NaCl, 10% glycerol, 5 mM dithiothreitol, 2 mM Na_2_MoO_4_, 2.5 mM NaF, 1.5 mM activated Na_3_VO_4_, 1 mM phenylmethanesulfonyl fluoride (PMSF), 1% IGEPAL, and cOmplete™ Protease Inhibitor Cocktail (Roche)). Immunoblotting was performed as previously described^26^.

For *Arabidopsis*, 7 days old seedlings were used for co-IP. Total proteins for each sample were incubated with 15 µl of GFP-Trap Agarose^®^ (GFP Nanobody coupled to agarose beads; Chromotek) 6 h in the cold room. GFP-Trap Agarose^®^ were washed with the co-IP buffer four times, then used for immunoblotting.

Anti-POR (1:1000), cpHSP70 (1:5000), and BRI1 (1:1000) antibodies were purchased from Agrisera (AS05 067, AS08 348, and AS12 1859). Anti-Flag (1:5000), α-Tubulin (1:5000), GST (1:5000), and MBP (1:5000) antibodies were purchased from Sigma-Aldrich (A8592, T6199, RPN1236, and M1321). Anti-GFP (1:1000) antibody was purchased from Miltenyi Biotec (130-091-833). Native DAY antibody, X-Q94C78-C (1:500), was purchased from AB-mart (http://www.ab-mart.com). X-Q94C78-C mouse monoclonal antibody gives raised with three amino acids from the C-terminal end of DAY protein sequence.

Peroxidase conjugated anti-Mouse (1:5000) and anti-Rabbit (1:10,000) secondary antibodies were purchased from Sigma-Aldrich (A9044 and A6154).

### Subcellular fractionation

Subcellular fractionation was performed as described^50^ with minor modification. 7-day old, light-grown seedlings were used for subcellular fractionation. After homogenization, 10 mL of isolation buffer (0.3 M sorbitol, 20 mM HEPES–KOH pH 8, 5 mM MgCl_2_, 5 mM EGTA, 5 mM EDTA) was added to the powder and vortexed for 10 s. Debris was removed using Miracloth and samples were centrifuged for 5 min at 4000 rpm. The resulting pellet was used for the isolation of intact chloroplasts. Pellets were resuspended in 2 mL isolation buffer and loaded onto 10 mL of a 30% percoll solution (percoll was diluted in isolation buffer) and centrifuged at 1800×*g* for 7 min. After elimination of the supernatant, the intact pellet was resuspended in 5 mL of import buffer (50 mM HEPES–KOH pH 8, 0.33 M sorbitol) and centrifuged at 1200×*g* for 5 min. The pellet was then dissolved in 1 mL import buffer and mixed with 5× SDS/PAGE sample buffer dye for further immunoblot analysis.

For isolation of a purified microsome fraction, 7-day old, light-grown seedlings were used. After homogenization, 2 mL of ice-cold sucrose buffer [20 mM Tris pH 8, 0.33 M Sucrose, 1 mM EDTA pH 8, protease inhibitor (Roche)] were added. Samples were centrifuged for 15 min at 5000×*g* at 4 °C. The supernatant was filtered through two layers of Miracloth and the centrifugation step was repeated until the supernatants were clear. The resulting total protein fractions were centrifuged at 4 °C for 60 min at 20,000×*g* to pellet microsomes. The pellet was resuspended in 100 µL of immunoprecipitation buffer (50 mM Tris pH 8, 150 mM NaCl, 1% Triton X-100) with extensive pipetting until the pellet was no longer visible and subsequently placed on a rotating wheel for 30 min at 4 °C. Samples were then pelleted for 10 min at 20,000×*g* at 4 °C. After this step, the supernatant was enriched in microsomal-associated proteins.

### Reverse transcription-quantitative PCR

Total RNA was extracted from seedlings or specific tissues using the Spectrum Plant Total RNA kit (Sigma Aldrich) or Universal RNA purification kit (Gene MATRIX). Total RNA was subjected to cDNA synthesis using the RevertAid First Strand cDNA synthesis kit (Thermo Fisher Scientific) or High Capacity cDNA Reverse Transcription Kit (Thermo Fisher Scientific) according to the manufacturer’s instructions. Reverse transcription-quantitative PCR was performed using the StepOnePlus Real-Time PCR system (Applied Biosystems) or LightCycler 96 (Roche) with SYBR green master mix. Transcription levels were normalized to that of PP2A A3 and are shown relative to the expression levels in indicated genotypes or treatment. Detailed information about primers is provided in Supplementary Data 2.

### Purification of recombinant proteins

Protein purification was performed as described^51^. DAY variants were cloned into a pMal-C2X vector (New England Biolabs) for MBP fusion and transformed into *E. coli* BL21 (DE3) cells. Cells were grown until they reached an OD600 between 0.6 and 0.8 in LB medium containing ampicillin (50 µg/mL) at 37 °C. 0.25 mM IPTG was then added and the cells were incubated for an additional 8 h for protein induction at 25 °C. MBP fusion proteins were purified with Amylose Resin (New England Biolabs) according to the manufacturer’s instructions with minor modification. A single buffer (20 mM Tris–HCl pH 7.5, 200 mM NaCl, 1% IGEPAL) was used throughout the purification procedure and proteins were eluted with elution buffer (20 mM Tris–HCl pH 7.5, 200 mM NaCl, 1% IGEPAL, 10 mM maltose). After purification, proteins were concentrated using Amicon Ultracel 30K (Millipore) according to the manufacturer’s instructions and maltose was removed by dialysis in either single buffer (20 mM Tris–HCl pH 7.5, 200 mM NaCl) or 50 mM HEPES–KOH pH 8.0 buffer for further analysis.

### In vitro binding assays

Bait proteins, immobilized on specific resin based on the corresponding protein tag, were incubated with *Escherichia coli* lysates expressing prey proteins in buffer (20 mM Tris–HCl pH 7.5, 200 mM NaCl, 10% glycerol, and 1% IGEPAL) for 1 h at room temperature. After washing four times (20 mM Tris–HCl pH 7.5, 200 mM NaCl, 10% glycerol, and 1% IGEPAL) on the resin, bound proteins were eluted with SDS sample buffer and subjected to immunoblotting.

### Measurement of chaperone activity

MDH (2 µM) was incubated in 50 mM HEPES–KOH pH 8.0 at 43 °C for 16 min with various concentrations of MBP-∆cTP DAY2 or 4 µM MBP-∆cTP/∆JL DAY or 10 µM BSA. Aggregation of MDH induced by heat was determined by following turbidity increase (*A*_340_) at 1-min intervals using a temperature-controlled spectrophotometer (DU800; Beckman).

### Virus-induced gene silencing

TRV2 vector carrying a fragment of DAY cDNA was used for VIGS in *Arabidopsis*. *Agrobacterium* harboring the TRV1 (pBINTRA) vector and *Agrobacterium* transformed with the cloned or empty TRV2 vector were cultured in LB medium supplemented with 10 mM MES–KOH pH 5.7 and 20 μM acetosyringone and grown overnight at 28 °C. Agrobacteria were harvested and resuspended in the infiltration buffer (10 mM MgCl_2_, 10 mM MES–KOH pH 5.7, and 200 μM acetosyringone). TRV2 and TRV1 agrobacteria were mixed at a 1:1 ratio with OD_600_ = 1 and incubated for 4 h, and then co-infiltrated into 10-day-old *Arabidopsis* seedlings. For *N. benthamiana* plants, VIGS was carried out with a similar method to *Arabidopsis* VIGS as described^26^. The sequence of *N. benthamiana* DAY is provided in Supplementary Data 3.

### Kinase assays

Kinase assays were performed as described^33^. Briefly, 40 μL reactions typically contained 0.1 mg/mL synthetic peptides, 1.0 μg of indicated proteins, and 0.1 mM [γ–^32^P]ATP in a buffer consisting of 50 mM MOPS [3-(*N*-morpholino) propane sulfonic acid], pH 7.5; 10 mM MgCl_2_, and 0.2 mM CaCl_2_. After 20 min incubation at 25 °C, 20 μL of the reaction was spotted on a 2 × 2 cm piece of P81 phosphocellulose paper. The paper was washed three times in 75 mM H_3_PO_4_ and then ^32^P incorporation into the peptide was determined by liquid scintillation counting. The Flag-BRI1 cytoplasmic domain was incubated with MBP or MBP-DAY 30 min before the assays. Kinase assays were performed with the BR13 peptide substrate (sequence: GRJKKIASVEJJK).

### Hypocotyl elongation measurement

These measurements were performed as described^52^. Briefly, sterilized seeds were plated on 1/2 MS with 1% sucrose and 0.8% phyto agar at 22 °C under long-day conditions. After 2 days of incubation at 4 °C, seedlings were irradiated with white light for 6 h to promote germination and then incubated in the dark. Hypocotyl lengths were measured using ImageJ software.

### Transcriptome analysis

RNA-seq was performed with Illumina Nextseq500. The Truseq-stranded mRNA prep kit (Illumina) was used for library preparation. The raw data were mapped and aligned with Tophat v2.0.13^53^, using the TAIR10 version of the *Arabidopsis* reference genome. Cuffdiff v2.2.0^54^ was used to identify differentially expressed genes (DEGs) using a threshold of fold change > 2. Gene set analysis was performed by calculating the enrichment of DEGs in Gene Ontology: Biological Process (GO:BP) terms^55^ with the Fisher’s exact test. The enrichment was considered significant if *P*-value ≤ 0.01 for each GO:BP term.

### Yeast two-hybrid

The cytosolic domain of BRI1 was introduced into pGBKT7. Two deletion constructs of DAY (∆cTP and ∆cTP/TM) were cloned into pGADT7. Various combinations of bait and prey plasmids were co-transformed into yeast (AH109). Yeast two-hybrid was performed according to the manufacturer’s instructions (Clontech). Interactions between BRI1 and DAY were defined by growth on SD/-Leu/-Trp/-His/-Ade media plates.

### Extracellular domain interaction assay

DAY deletion variants and the extracellular domain of BRI1 were expressed in S2 cells. DAY variants and the extracellular domain of BRI1 were cloned into pECIA2 as a prey tagged with AP and pECIA14 as a bait tagged with Fc, respectively. Protein A-coated wells were washed with phosphate-buffered saline (PBS) containing 0.1% Tween-20, and Fc-fusion proteins (bait) and AP-fusion proteins (prey) were then added prior to incubation overnight at 4 °C. Absorbance at 650 nm was measured at 2 h using a BioTek Synergy 4 with gen5 2.08 software by adding the BluePhos Phosphatase Substrate (Seracare Life Sciences) to each well.

### Reporting summary

Further information on research design is available in the Nature Research Reporting Summary linked to this article.

## Supplementary information

Supplementary Information

Descriptions of Additional Supplementary Files

Supplementary data 1

Supplementary data 2

Supplementary data 3

Reporting Summary

## Data Availability

RNA sequencing data were deposited into the Gene Expression Omnibus database under accession number GSE177028 and the NCBI Sequence Read Archive under accession number SRP323708. Arabidopsis mutants and transgenic lines used in this study are available from the corresponding author upon reasonable request. The source data for all figures and Supplementary figures can be found in the Source Data file. Source data are provided with this paper.

## Source data

Source Data

## References

[1] Xu, X., Paik, I., Zhu, L. & Huq, E. Illuminating progress in phytochrome-mediated light signaling pathways. *Trends Plant Sci.*10.1016/j.tplants.2015.06.010 (2015). 10.1016/j.tplants.2015.06.010 26440433

[2] Wu, S.-H. Gene expression regulation in photomorphogenesis from the perspective of the Central Dogma. *Annu. Rev. Plant Biol*. 10.1146/annurev-arplant-050213-040337 (2014). 10.1146/annurev-arplant-050213-040337 24779996

[3] Reinbothe, C. et al. Chlorophyll biosynthesis: spotlight on protochlorophyllide reduction. *Trends Plant Sci.*10.1016/j.tplants.2010.07.002 (2010). 10.1016/j.tplants.2010.07.002 20801074

[4] Wang, Z.-Y., Bai, M.-Y., Oh, E. & Zhu, J.-Y. Brassinosteroid signaling network and regulation of photomorphogenesis. *Annu. Rev. Genet*. 10.1146/annurev-genet-102209-163450 (2011). 10.1146/annurev-genet-102209-163450 23020777

[5] Singh, A. P. & Savaldi-Goldstein, S. Growth control: brassinosteroid activity gets context. *J. Exp. Bot.*10.1093/jxb/erv026 (2015). 10.1093/jxb/erv026 25673814

[6] Chory, J., Nagpal, P. & Peto, C. A. Phenotypic and genetic analysis of det2, a New mutant that affects light-regulated seedling development in *Arabidopsis*. *Plant Cell*10.2307/3869351 (2007). 10.1105/tpc.3.5.445 PMC160013 12324600

[7] Clouse, S. D., Langford, M. & McMorris, T. C. A brassinosteroid-insensitive mutant in *Arabidopsis thaliana* exhibits multiple defects in growth and development. *Plant Physiol*. 10.1104/pp.111.3.671 (2002). 10.1104/pp.111.3.671 PMC157882 8754677

[8] Li, J., Nagpal, P., Vitart, V., McMorris, T. C. & Chory, J. A role for brassinosteroids in light-dependent development of *Arabidopsis*. *Science (80-.)*. 10.1126/science.272.5260.398 (1996). 10.1126/science.272.5260.398 8602526

[9] Belkhadir, Y. & Jaillais, Y. The molecular circuitry of brassinosteroid signaling. *New Phytol*. 10.1111/nph.13269 (2015). 10.1111/nph.13269 25615890

[10] Russinova, E. et al. The clathrin adaptor complex AP-2 mediates endocytosis of BRASSINOSTEROID INSENSITIVE1 in *Arabidopsis*. *Plant Cell*10.1105/tpc.113.114058 (2013). 10.1105/tpc.113.114058 PMC3784593 23975899

[11] Zhou, J. et al. Regulation of Arabidopsis brassinosteroid receptor BRI1 endocytosis and degradation by plant U-box PUB12/PUB13-mediated ubiquitination. *Proc. Natl. Acad. Sci*. *USA*10.1073/pnas.1712251115 (2018). 10.1073/pnas.1712251115 PMC5828578 29432171

[12] Hong, Z., Jin, H., Tzfira, T. & Li, J. Multiple mechanism-mediated retention of a defective brassinosteroid receptor in the endoplasmic reticulum of *Arabidopsis*. *PLANT CELL ONLINE*10.1105/tpc.108.061879 (2008). 10.1105/tpc.108.061879 PMC2630446 19060110

[13] Irani, N. G. et al. Fluorescent castasterone reveals BRI1 signaling from the plasma membrane. *Nat. Chem. Biol*. 10.1038/nchembio.958 (2012). 10.1038/nchembio.958 22561410

[14] Martins, S. et al. Internalization and vacuolar targeting of the brassinosteroid hormone receptor BRI1 are regulated by ubiquitination. *Nat. Commun*. 10.1038/ncomms7151 (2015). 10.1038/ncomms7151 PMC4713032 25608221

[15] Geldner, N., Hyman, D. L., Wang, X., Schumacher, K. & Chory, J. Endosomal signaling of plant steroid receptor kinase BRI1. *Genes Dev*. 10.1101/gad.1561307 (2007). 10.1101/gad.1561307 PMC1899468 17578906

[16] Yin, Y. et al. BES1 accumulates in the nucleus in response to brassinosteroids to regulate gene expression and promote stem elongation. *Cell*. **109**, 181–191 (2002). 10.1016/s0092-8674(02)00721-3 12007405

[17] Wang, Z. Y. et al. Nuclear-localized BZR1 mediates brassinosteroid-induced growth and feedback suppression of brassinosteroid biosynthesis. *Dev. Cell*10.1016/S1534-5807(02)00153-3 (2002). 10.1016/s1534-5807(02)00153-3 11970900

[18] He, J.-X., Gendron, J. M., Yang, Y., Li, J. & Wang, Z.-Y. The GSK3-like kinase BIN2 phosphorylates and destabilizes BZR1, a positive regulator of the brassinosteroid signaling pathway in *Arabidopsis*. *Proc. Natl. Acad. Sci. USA*10.1073/pnas.152342599 (2002). 10.1073/pnas.152342599 PMC126645 12114546

[19] Sun, Y. et al. Integration of Brassinosteroid signal transduction with the transcription network for plant growth regulation in *Arabidopsis*. *Dev. Cell*10.1016/j.devcel.2010.10.010 (2010). 10.1016/j.devcel.2010.10.010 PMC3018842 21074725

[20] Oh, E., Zhu, J. Y. & Wang, Z. Y. Interaction between BZR1 and PIF4 integrates brassinosteroid and environmental responses. *Nat. Cell Biol*. 10.1038/ncb2545 (2012). 10.1038/ncb2545 PMC3703456 22820378

[21] Pedmale, U. V. et al. Cryptochromes interact directly with PIFs to control plant growth in limiting blue light. *Cell*10.1016/j.cell.2015.12.018 (2016). 10.1016/j.cell.2015.12.018 PMC4721562 26724867

[22] Lau, O. S. & Deng, X. W. The photomorphogenic repressors COP1 and DET1: 20 years later. *Trends Plant Sci.*10.1016/j.tplants.2012.05.004 (2012). 10.1016/j.tplants.2012.05.004 22705257

[23] Liu, H., Liu, B., Zhao, C., Pepper, M. & Lin, C. The action mechanisms of plant cryptochromes. *Trends Plant Sci.*10.1016/j.tplants.2011.09.002 (2011). 10.1016/j.tplants.2011.09.002 PMC3277817 21983106

[24] Reinbothe, S., Reinbothe, C., Holtorf, H. & Apel, K. Two NADPH:protochlorophyllide oxidoreductases in Barley: evidence for the selective disappearance of PORA during the light-induced greening of etiolated seedlings. *Plant Cell*10.2307/3870200 (2007). 10.1105/tpc.7.11.1933 PMC161051 12242364

[25] Masuda, T. & Takamiya, K. I. Novel insights into the enzymology, regulation and physiological functions of light-dependent protochlorophyllide oxidoreductase in angiosperms. *Photosynth. Res.*10.1023/B:PRES.0000028392.80354.7c (2004). 10.1023/B:PRES.0000028392.80354.7c 16328844

[26] Lee, J.-Y. et al. Cell growth defect Factor1/CHAPERONE-LIKE PROTEIN OF POR1 plays a role in stabilization of light-dependent protochlorophyllide oxidoreductase in *Nicotiana benthamiana* and *Arabidopsis*. *Plant Cell*10.1105/tpc.113.111096 (2013). 10.1105/tpc.113.111096 PMC3877821 24151298

[27] Reinbothe, S., Gray, J., Rustgi, S., von Wettstein, D. & Reinbothe, C. Cell growth defect factor 1 is crucial for the plastid import of NADPH:protochlorophyllide oxidoreductase A in *Arabidopsis thaliana*. *Proc. Natl. Acad. Sci*. *USA*10.1073/pnas.1506339112 (2015). 10.1073/pnas.1506339112 PMC4426407 25901327

[28] Kawai-Yamada, M. et al. A novel arabidopsis gene causes Bax-like lethality in *Saccharomyces cerevisiae*. *J. Biol. Chem*. 10.1074/jbc.M509632200 (2005). 10.1074/jbc.M509632200 16192270

[29] Cui, M. H. et al. An arabidopsis cell growth defect factor-related protein, CRS, promotes plant senescence by increasing the production of hydrogen peroxide. *Plant Cell Physiol*. 10.1093/pcp/pcs161 (2013). 10.1093/pcp/pcs161 23220690

[30] Rajan, V. B. V. & D’Silva, P. *Arabidopsis thaliana* J-class heat shock proteins: cellular stress sensors. *Funct. Integr. Genom.*10.1007/s10142-009-0132-0 (2009). 10.1007/s10142-009-0132-0 19633874

[31] Qiu, X. B., Shao, Y. M., Miao, S. & Wang, L. The diversity of the DnaJ/Hsp40 family, the crucial partners for Hsp70 chaperones. *Cell. Mol. Life Sci.*10.1007/s00018-006-6192-6 (2006). 10.1007/s00018-006-6192-6 PMC11136209 16952052

[32] Oh, M.-H. et al. Recombinant Brassinosteroid Insensitive 1 receptor-like kinase autophosphorylates on serine and threonine residues and phosphorylates a conserved peptide motif in vitro. *Plant Physiol*. 10.1104/pp.124.2.751 (2000). 10.1104/pp.124.2.751 PMC59180 11027724

[33] Oh, M.-H., Clouse, S. D. & Huber, S. C. Tyrosine phosphorylation of the BRI1 receptor kinase occurs via a post-translational modification and is activated by the juxtamembrane domain. *Front. Plant Sci*. 10.3389/fpls.2012.00175(2012). 10.3389/fpls.2012.00175 PMC3413876 22891071

[34] Zhang, X. et al. A temperature-sensitive misfolded bri1-301 receptor requires its kinase activity to promote growth 1[open]. *Plant Physiol*. 10.1104/pp.18.00452 (2018). 10.1104/pp.18.00452 PMC6288740 30333151

[35] Smakowska-Luzan, E. et al. An extracellular network of *Arabidopsis* leucine-rich repeat receptor kinases. *Nature*10.1038/nature25184 (2018). 10.1038/nature25184 PMC6485605 29320478

[36] Baslam, M., Oikawa, K., Kitajima-Koga, A., Kaneko, K. & Mitsui, T. Golgi-to-plastid trafficking of proteins through secretory pathway: insights into vesicle-mediated import toward the plastids. *Plant Signal. Behav*. 10.1080/15592324.2016.1221558 (2016). 10.1080/15592324.2016.1221558 PMC5058459 27700755

[37] Villarejo, A. et al. Evidence for a protein transported through the secretory pathway en route to the higher plant chloroplast. *Nat. Cell Biol*. 10.1038/ncb1330 (2005). 10.1038/ncb1330 16284624

[38] Caplan, A. J., Mandal, A. K. & Theodoraki, M. A. Molecular chaperones and protein kinase quality control. *Trends Cell Biol.*10.1016/j.tcb.2006.12.002 (2007). 10.1016/j.tcb.2006.12.002 17184992

[39] Prince, T. & Matts, R. L. Definition of protein kinase sequence motifs that trigger high affinity binding of Hsp90 and Cdc37. *J. Biol. Chem*. 10.1074/jbc.M406882200 (2004). 10.1074/jbc.M406882200 15258137

[40] Prince, T. & Matts, R. L. Exposure of protein kinase motifs that trigger binding of Hsp90 and Cdc37. *Biochem. Biophys. Res. Commun*. 10.1016/j.bbrc.2005.10.100 (2005). 10.1016/j.bbrc.2005.10.100 16269130

[41] Bojar, D. et al. Crystal structures of the phosphorylated BRI1 kinase domain and implications for brassinosteroid signal initiation. *Plant J*. 10.1111/tpj.12445 (2014). 10.1111/tpj.12445 PMC4260089 24461462

[42] Li, C. et al. Glycosylphosphatidylinositol-anchored proteins as chaperones and co-receptors for FERONIA receptor kinase signaling in Arabidopsis. *Elife*10.7554/eLife.06587 (2015). 10.7554/eLife.06587 PMC4458842 26052747

[43] Chen, M. et al. Arabidopsis HEMERA/pTAC12 Initiates photomorphogenesis by phytochromes. *Cell*10.1016/j.cell.2010.05.007 (2010). 10.1016/j.cell.2010.05.007 PMC2935685 20603003

[44] Galvão, R. M. et al. Photoactivated phytochromes interact with HEMERA and promote its accumulation to establish photomorphogenesis in *Arabidopsis*. *Genes Dev*. 10.1101/gad.193219.112 (2012) 10.1101/gad.193219.112 PMC3426763 22895253

[45] Qiu, Y. et al. Hemera couples the proteolysis and transcriptional activity of phytochrome interacting actors in arabidopsis photomorphogenesis. *Plant Cell*10.1105/tpc.114.136093 (2015). 10.1105/tpc.114.136093 PMC4456642 25944101

[46] Nevarez, P. A. et al. Mechanism of dual targeting of the phytochrome signaling component HEMERA/pTAC12 to plastids and the nucleus. *Plant Physiol*. 10.1104/pp.16.00116 (2017). 10.1104/pp.16.00116 PMC5373053 28232584

[47] Qiu, Y., Li, M., Kim, R. J. A., Moore, C. M. & Chen, M. Daytime temperature is sensed by phytochrome B in *Arabidopsis* through a transcriptional activator HEMERA. *Nat. Commun*. 10.1038/s41467-018-08059-z (2019). 10.1038/s41467-018-08059-z PMC6329817 30635559

[48] Ma, L. et al. Light control of *Arabidopsis* development entails coordinated regulation of genome expression and cellular pathways. *Plant Cell*10.2307/3871521 (2001). 10.1105/tpc.010229 PMC139475 11752374

[49] Carrie, C. & Small, I. A reevaluation of dual-targeting of proteins to mitochondria and chloroplasts. *Biochim. Biophys. Acta*10.1016/j.bbamcr.2012.05.029 (2013). 10.1016/j.bbamcr.2012.05.029 22683762

[50] Mehrshahi, P. et al. Transorganellar complementation redefines the biochemical continuity of endoplasmic reticulum and chloroplasts. *Proc. Natl. Acad. Sci. USA*10.1073/pnas.1306331110 (2013). 10.1073/pnas.1306331110 PMC3718160 23818635

[51] Lee, D. H., Park, S. J., Ahn, C. S. & Pai, H. S. MRF family genes are involved in translation control, especially under energy-deficient conditions, and their expression and functions are modulated by the TOR signaling pathway. *Plant Cell*10.1105/tpc.17.00563 (2017). 10.1105/tpc.17.00563 PMC5728134 29084871

[52] Belkhadir, Y. et al. Brassinosteroids modulate the efficiency of plant immune responses to microbe-associated molecular patterns. *Proc. Natl. Acad. Sci. USA*10.1073/pnas.1112840108 (2012). 10.1073/pnas.1112840108 PMC3252953 22087001

[53] Trapnell, C., Pachter, L. & Salzberg, S. L. TopHat: discovering splice junctions with RNA-Seq. *Bioinformatics*10.1093/bioinformatics/btp120 (2009). 10.1093/bioinformatics/btp120 PMC2672628 19289445

[54] Trapnell, C. et al. Transcript assembly and quantification by RNA-Seq reveals unannotated transcripts and isoform switching during cell differentiation. *Nat. Biotechnol*. 10.1038/nbt.1621 (2010). 10.1038/nbt.1621 PMC3146043 20436464

[55] Blake, J. A. et al. Gene ontology consortium: going forward. *Nucleic Acids Res*. 10.1093/nar/gku1179 (2015). 10.1093/nar/gku1179 PMC4383973 25428369

