# Supplementary material for: Chaperone-like protein DAY plays critical roles in photomorphogenesis
Source: Nat Commun. 2021 Jul 7;12:4194. doi: 10.1038/s41467-021-24446-5 (PMC8263706; doi:10.1038/s41467-021-24446-5)
Supplement: Supplementary file 1 — Supplementary Information [file 41467_2021_24446_MOESM1_ESM.pdf]

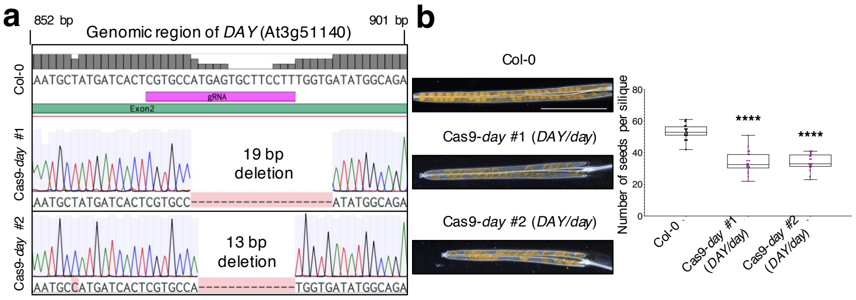

c

Cas9-*day* #1 (plant numbers)					Cas9-*day* #1 (%)			
Plant	*day / day*	*DAY / DAY*	*DAY / day*	Dead seedlings	*day / day*	*DAY / DAY*	*DAY / day*	Dead seedlings
#1	0	10	9	13	0.00	31.25	28.13	40.63
#2	0	14	12	22	0.00	29.17	25.00	45.83
Cas9-*day* #2 (plant numbers)					Cas9-*day* #2 (%)			
Plant	*day / day*	*DAY / DAY*	*DAY / day*	Dead seedlings	*day / day*	*DAY / DAY*	*DAY / day*	Dead seedlings
#1	0	12	8	12	0.00	37.50	25.00	37.50
#2	0	15	13	20	0.00	31.25	27.08	41.67

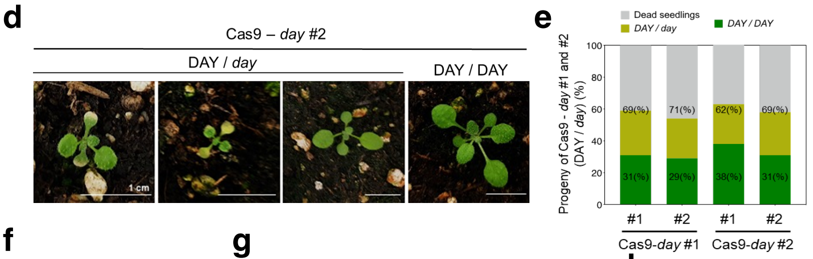

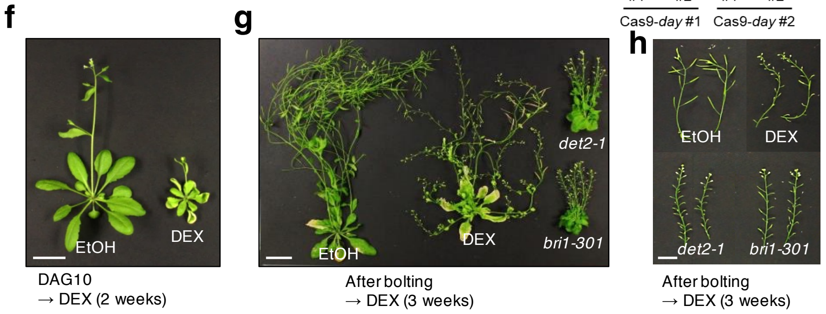

Supplementary Fig. 1 Characterization of CRISPR/Cas9-*day* mutant lines and analyses of the phenotypes after knockdown of *DAY*. a, The overview of CRISPR/Cas9-mediated *day* mutant (Cas9-*day*) #1 and #2 lines. The panel shows the sequence chromatograms taken from Benchling. **b**, Seeds counting of CRISPR/Cas9-*day* mutants. The left panel shows representative pictures of siliques after clearing with Hoyer's solution. Scale bar: 1 cm. The right panel shows quantification of seed numbers per silique. Dots represent individual observations. Twenty siliques were examined for each genotype. Statistical significance was determined by one-way ANOVA, Dunnett's multiple comparisons test ****, P < 0.0001. **c**, Progeny analysis of CRISPR/Cas9-*day* mutants. Notably, the *day/day* genotype was missing among the progeny. **d**. Representative phenotypes of the *DAY/day* heterozygous mutant plants, compared with the *DAY/DAY* phenotypes. **e**. The abundance of *DAY/DAY*, *DAY/day*, and perished seedlings in the progeny of Cas9-day (*DAY/day*) was quantified as a percentage of their total numbers. The % values in the middle of the graph are the total of the percentages of the *DAY/day* and perished seedlings. **f**, **g**, *Arabidopsis* Dexamethasone (DEX)-inducible *DAY* RNAi plants (*DAYi*) exhibit growth retardation, leaf yellowing, short petioles, and retarded silique growth upon DEX treatment. Plants were grown in soil for 10 days (**f**) or until bolting (**g**), and were then sprayed with either ethanol or 1 μM DEX for two (**f**) or three weeks (**g**). Scale bar, 3 cm. **h**, Morphology of silique growth in *DAYi* plants mimics those of BR biosynthesis (*det2-1*) and receptor (*bri1-301*) mutants. Scale bar: 1 cm.

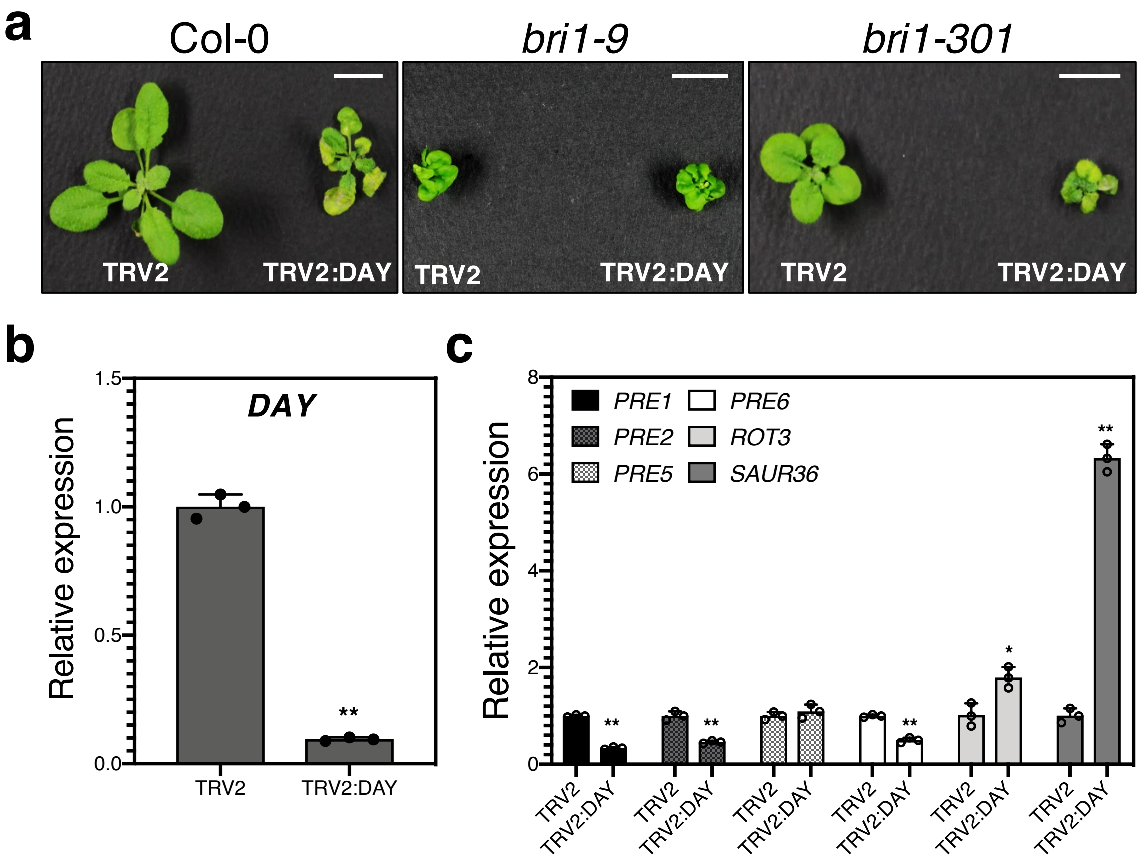

Supplementary Fig. 2 Reduction of *DAY* in Col-0 and *bri1-301*, but not *bri1-9*, caused growth retardation and leaf yellowing. a, Phenotypes of *DAY* VIGS in *Arabidopsis*. *Arabidopsis* WT (Col-0; 6-leaf stage), *bri1-9* (4-leaf stage) and *bri1-301* (4-leaf stage) plants were subjected to VIGS. The *bri1-9* allele carries a mutation in the BRI1 extracellular domain (S662F), and *bri1-301* is a weak mutant allele with a mutation in the kinase domain (G989I) [1,2]. VIGS-mediated knockdown of *DAY* in the WT and *bri1-301* backgrounds, but not in *bri1-9*, caused growth retardation, including extremely short petioles and leaf yellowing, when compared with the VIGS vector control (TRV2). For VIGS, the *DAY* cDNA fragment (436 bp; nucleotide residues 401–837) was cloned into the VIGS vector pTRV2 and *Arabidopsis* plants were infiltrated with Agrobacterium containing the TRV2 or TRV2:DAY constructs. Plants were photographed 2 weeks after infiltration. Scale bar, 1 cm. **b**, **c**, RT-qPCR analysis of *DAY* transcript levels (**b**) and BR-related genes (**c**) in *DAY* VIGS plants. The expression levels of the BR-induced genes *PRE1*, *PRE2,* and *PRE6* decreased, while expression of the BR-repressed genes *ROT3* and *SAUR36* increased. Transcript levels were normalized to *PP2A A3* mRNA and expressed relative to those of the TRV2 control. Error bars indicate SD from three technical replicates. Asterisks denote statistical significance of the differences between TRV2 and TRV2:DAY based on unpaired, two-tailed Student's t-test. *, $P \leq 0.05$ and **, $P \leq 0.01$.

1. Noguchi, T. *et al.* Brassinosteroid-Insensitive Dwarf Mutants of Arabidopsis Accumulate Brassinosteroids. *Plant Physiol.* (1999). doi:10.1104/pp.121.3.743

2. Xu, W., Huang, J., Li, B., Li, J. & Wang, Y. Is kinase activity essential for biological functions of BRI1? *Cell Res.* (2008). doi:10.1038/cr.2008.36

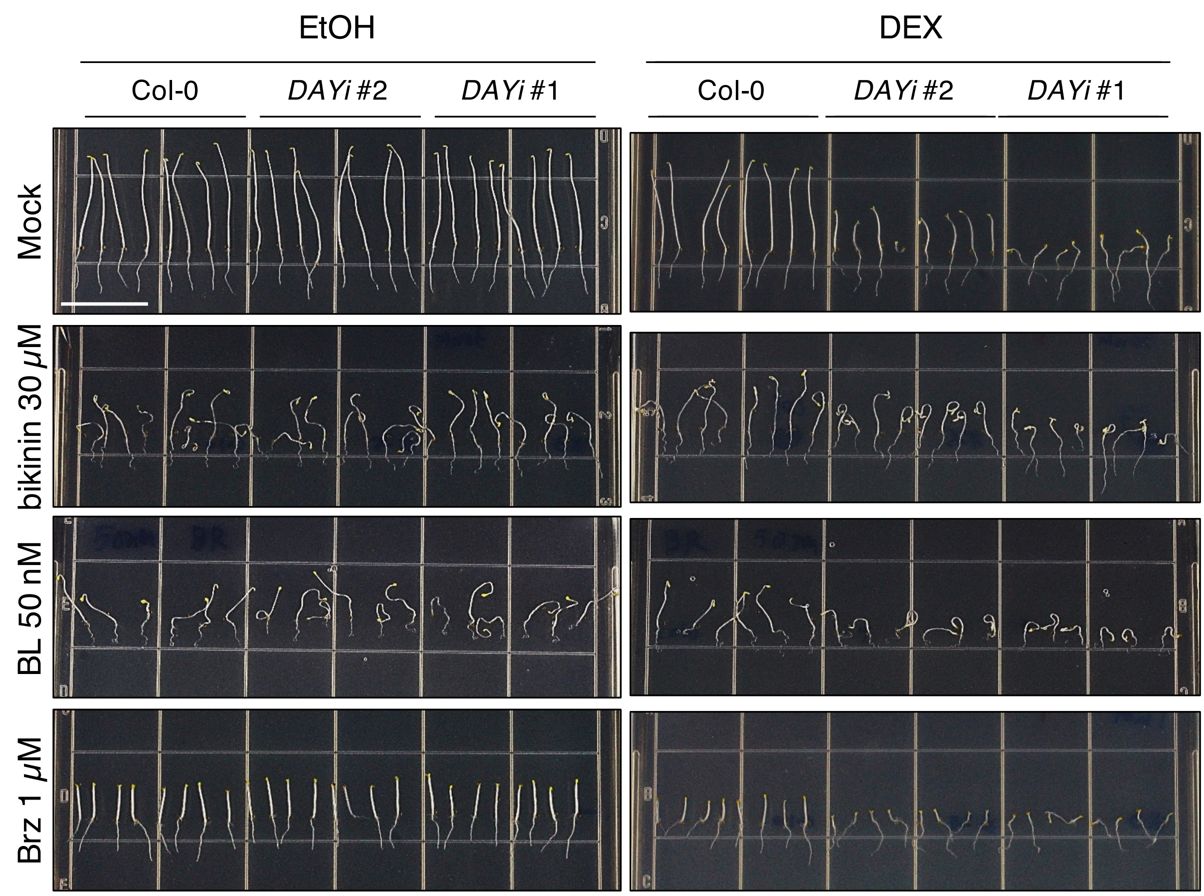

Supplementary Fig. 3 *DAY* promotes BR signaling in the dark. Seedlings were grown in the dark for 5 days on medium containing ethanol (EtOH) or 1 μM DEX (DEX) with 30 μM bikinin, 50 nM BL, or 1 μM BR biosynthesis inhibitor brassinazole (Brz). Scale bar for all pictures, 2 cm.

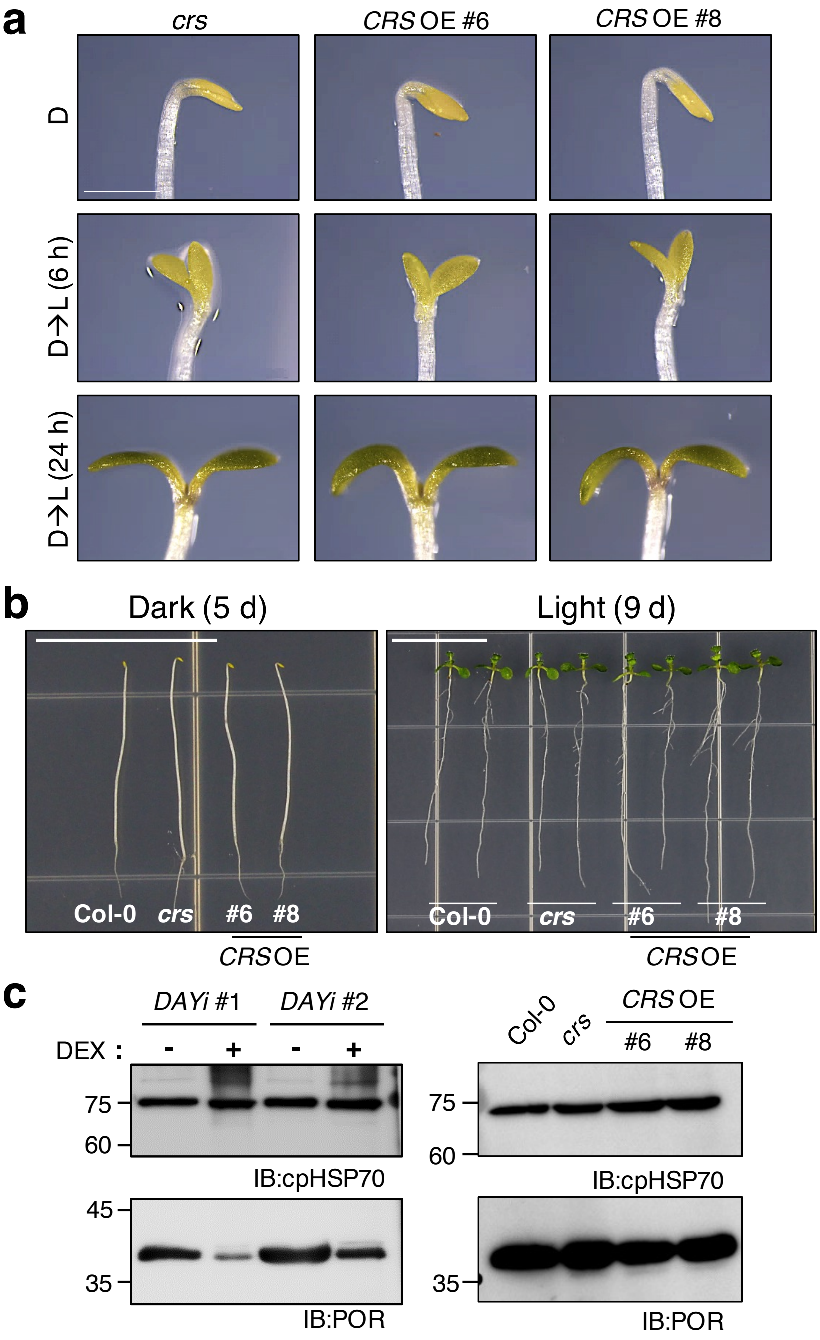

Supplementary Fig. 4 The *DAY* homolog, *CDF-RELATED GENE RESPONSIVE TO SENESCENCE (CRS),* is not required for light-induced cotyledon greening. a, The *crs* mutant and *CRS* overexpression (OE) plants (line #6 and #8) show normal cotyledon greening during the dark-to-light transition. Seedlings were grown on ½ MS medium in the dark for 5 days (D), and then transferred to the light for 6 or 24 h (L). Scale bar for all pictures, 0.1 cm. **b**, *crs* mutant and *CRS* OE plants show normal growth in the dark (left panel) and in the light (right panel). Seedlings were grown on ½ MS medium in the dark for 5 days or in the light for 9 days. Scale bar, 2 cm. **c,** DEX-treated *DAYi*, but not the *crs* mutation or *CRS* OE, show a decrease in POR protein accumulation. *DAYi* seedlings (line #1 and #2) were grown in the dark for 5 days on ½ MS medium containing either ethanol (-) or 1 µM DEX (+), and subjected to immunoblotting using antibodies against cpHSP70 and POR (left panel). The *crs* and *CRS* OE seedlings were grown on ½ MS medium in the dark for 5 days before immunoblotting (right panel).

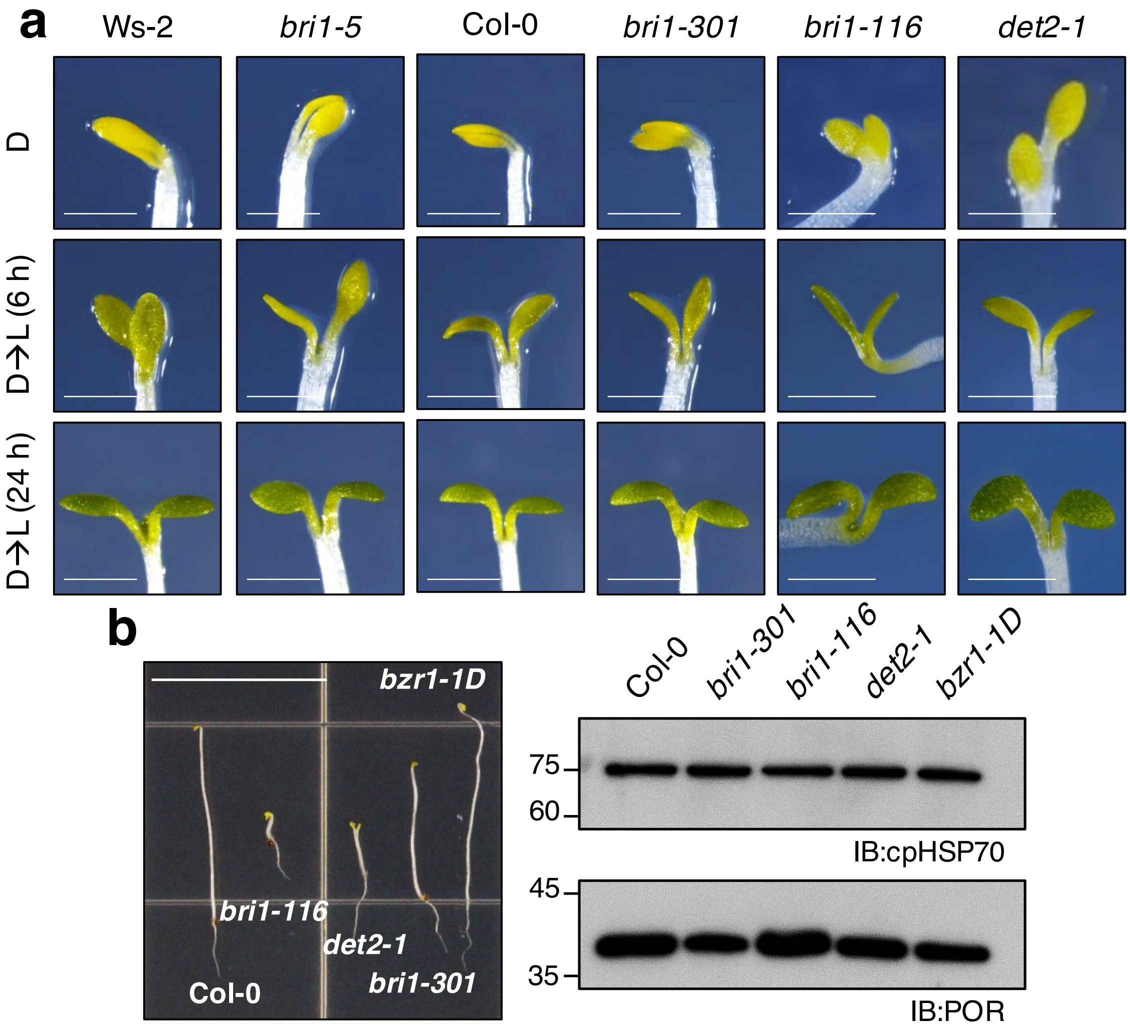

Supplementary Fig. 5 Mutations in BR signaling or biosynthesis do not affect cotyledon greening and POR protein accumulation in the light and dark, respectively. **a**, Seedlings of the WT (Ws-2 and Col-0), BR receptor mutants (*bri1-5*, *bri1-301*, and *bri1-116*), and BR biosynthetic mutant (*det2-1*) were grown on ½ MS medium in the dark for 5 days (D), and then exposed to light (L) for 6 or 24 h. Scale bar, 0.1 cm. **b**, Seedlings of the indicated genotypes were grown on ½ MS medium in the dark for 5 days (left panel), and then subjected to immunoblotting with anti-cpHSP70 and anti-POR antibodies (right panel). POR protein levels were mostly consistent across all genotypes. *bzr1-1D*, *brassinazole-resistant 1-1D.* Scale bar, 2 cm.

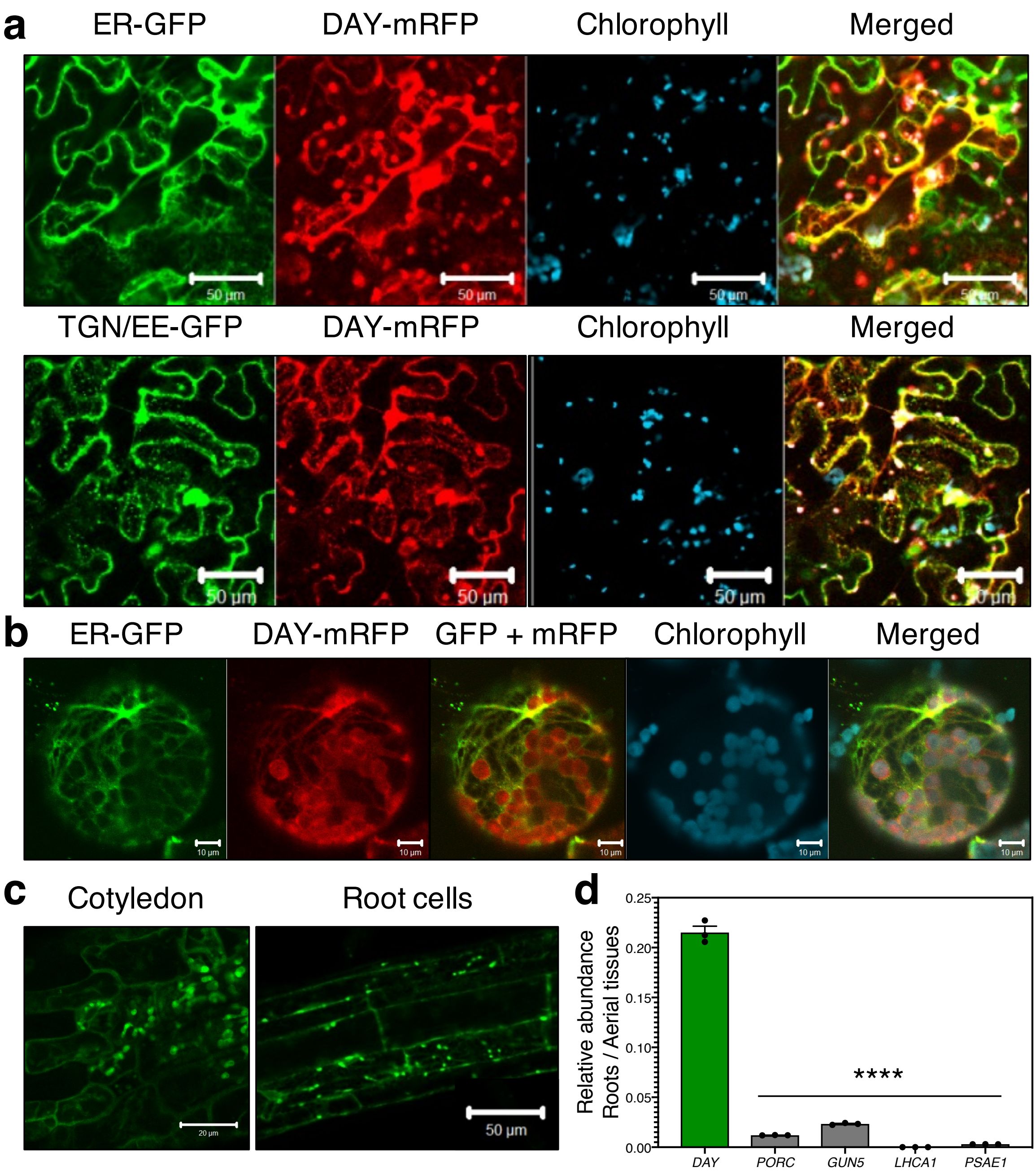

Supplementary Fig. 6 DAY is localized to chloroplasts, ER, TGN/EE and plasma membrane. a, mRFP and GFP fusion proteins were transiently co-expressed in *N. benthamiana* leaves by agroinfiltration and epidermal layers were observed by CLSM. ER-GFP or TGN/EE-GFP were used as an ER or TGN/EE marker, respectively. Scale bar, 50 μm. **b,** DAY-mRFP co-localizes with ER-GFP and chlorophyll auto-fluorescence in *N. benthamiana* mesophyll protoplasts. Scale bar, 10 μm. **c**, GFP signals from in transgenic *Arabidopsis* plants expressing DAY-GFP under the CaMV35S promoter (35S::DAY-GFP). DAY-GFP is localized to the plasma membrane, mesh-like structures, and inner cell speckles in dark-grown cotyledons and light-grown root cells. Scale bar, 20 μm (cotyledon) or 50 μm (root). **d,** *DAY* mRNA levels are significantly higher than other chloroplast-related genes in roots. RT-qPCR was performed to determine transcript levels of chloroplast-related genes and *DAY* in aerial tissues and roots. Transcript levels were normalized to *PP2A A3* mRNA. Error bars indicate SD from three technical replicates. Statistical significance was determined by one-way ANOVA, Dunnett's multiple comparisons test ****, $P < 0.0001$.

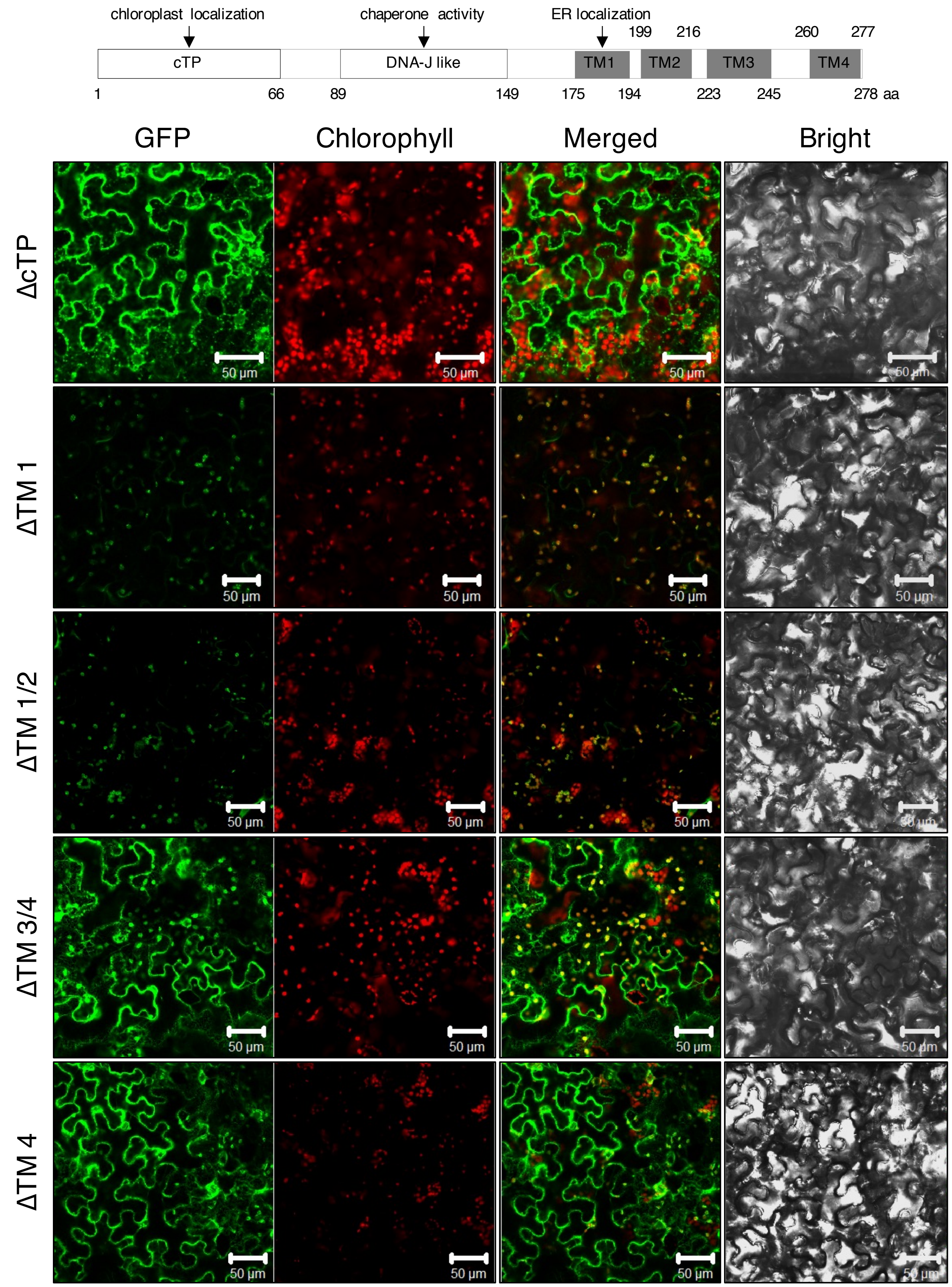

Supplementary Fig. 7 The cTP and TM1 are important for targeting of DAY to chloroplasts and the endomembrane system, respectively. Green fluorescence of the indicated DAY-GFP variants was merged with chloroplast autofluorescence (Chlorophyll) in epidermal cells. Constructs were expressed in *N. benthamiana* leaves via agroinfiltration. ΔcTP, deletion of cTP; ΔTM1, deletion of TM1; ΔTM1/2, deletion of TM1 and TM2; ΔTM3/4, deletion of TM3 and TM4; ΔTM4, deletion of TM4. Scale bar, 50 μm.

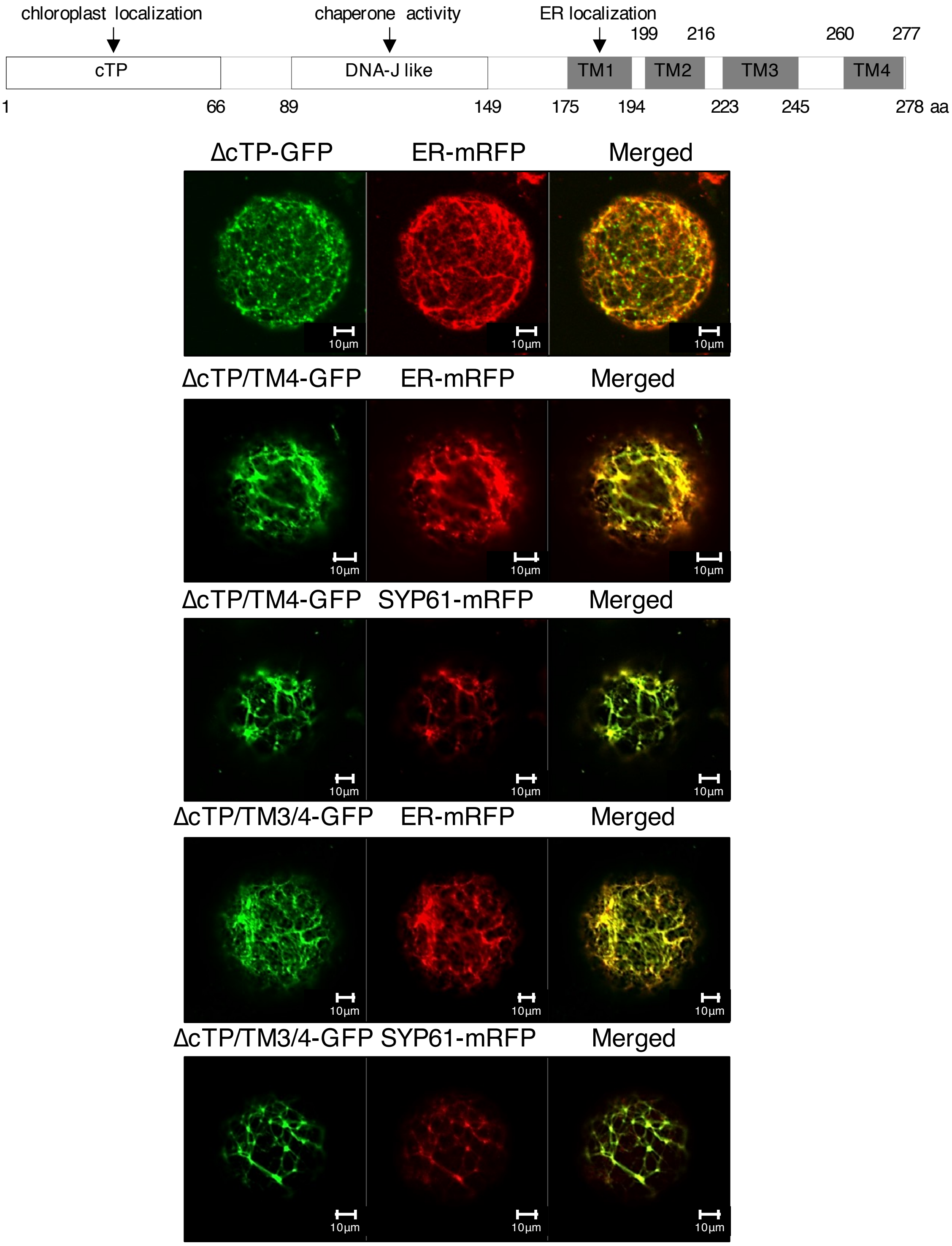

Supplementary Fig. 8 The cTP, TM3, and TM4 are not required for DAY localization to the endomembrane system. Green fluorescence of the indicated DAY-GFP variants was merged with ER-mRFP or SYP61-mRFP in leaf protoplasts. Constructs were expressed in *N. benthamiana* leaves via agroinfiltration, and leaf protoplasts were generated for observation under CLSM. ΔcTP, deletion of cTP; ΔTM3/4, deletion of TM3 and TM4; ΔTM4, deletion of TM4. Scale bar, 10 μm.

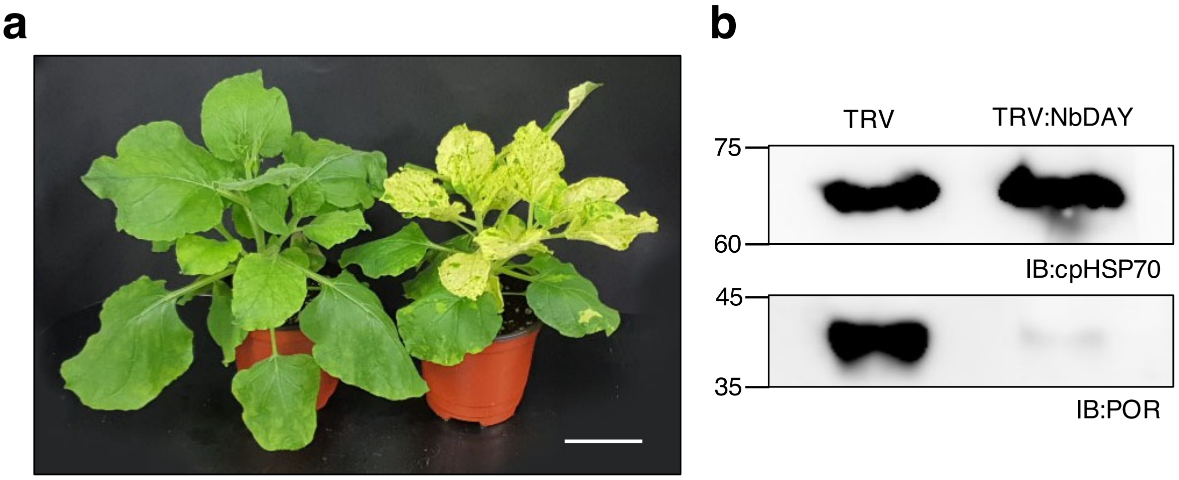

Supplementary Fig. 9 Reduction of *DAY* causes leaf yellowing and growth retardation in *N. benthamiana*. Related to Fig. 3a. a, 6-leaf stage *N. benthamiana* plants were subjected to VIGS using the tobacco rattle virus (TRV) system. VIGS of Nb*DAY* (TRV:NbDAY) and TRV control plants were photographed 4 weeks after infiltration. Scale bar, 5 cm. **b**, Immunoblot analyses of TRV:NbDAY and TRV control plants indicating that *DAY* promotes the accumulation of POR in the light.

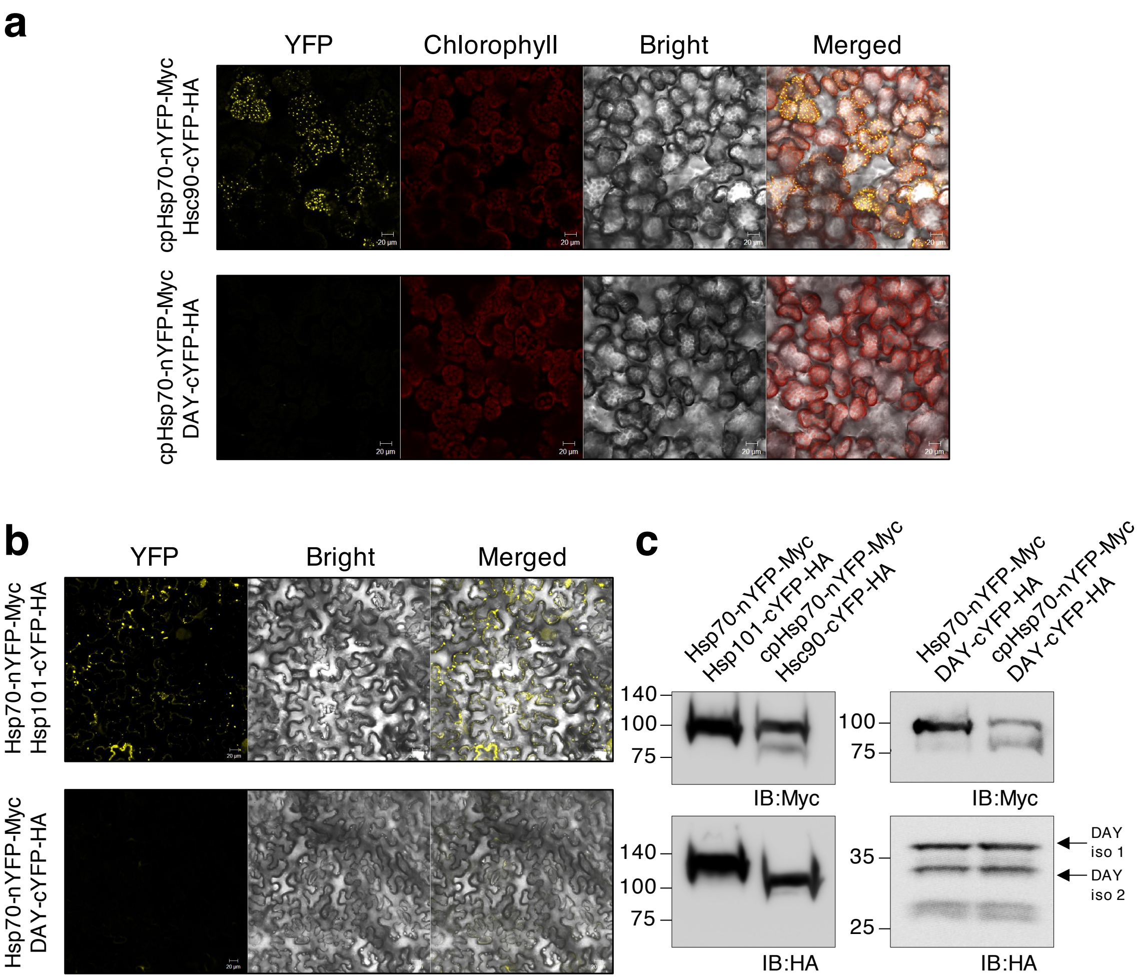

Supplementary Fig. 10 DAY interacts with neither chloroplast Hsp70 (cpHsp70) nor cytosolic Hsp70 (Hsp70). a, **b**, BiFC assays between DAY and cpHsp70 (**a**) or Hsp70 (**b**). YFP signals were examined by CLSM in mesophyll cells (**a**) or epidermal cells (**b**) after agroinfiltration. Interactions between cpHsp70 and Hsc90 in chloroplasts (**a**), and between Hsp70 and Hsp101 in the cytosol (**b**) were observed as positive controls. Scale bar, 20 μm. **c**, Immunoblotting using anti-Myc and anti-HA antibodies to examine protein expression in (**a**) and (**b**).

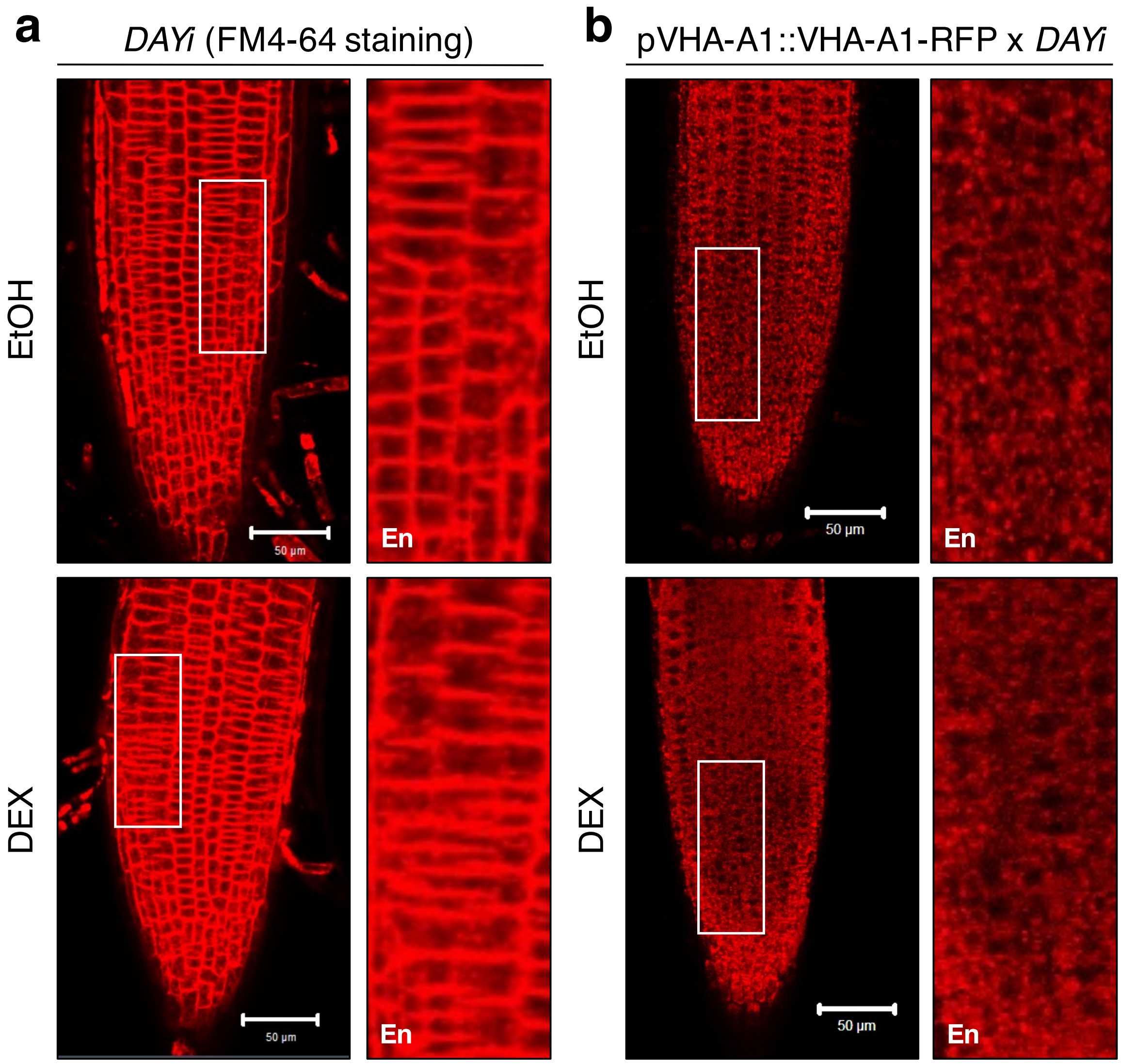

Supplementary Fig. 11 DAY deficiency affects neither endocytosis nor VHA-A1 distribution in *Arabidopsis* roots. *DAYi* (**a**) and *DAYi* x pVHA-A1::VHA-A1-RFP (**b**) seedlings were grown in the light for 9 days in the presence of ethanol or 1 μM DEX and root tips were observed by CLSM. To visualize endocytic vesicles, roots were stained with FM4-64 dye for 30 min before CLSM. To visualize TGN/EE, *DAYi* plants were crossed with transgenic plants expressing an RFP fusion protein of VHA-A1 (pVHA-A1::VHA-A1-RFP). Enlarged views of the *boxed* areas are shown (*En*). VHA-A1,VACUOLAR H^+ ATPASE A1 SUBUNIT. Scale bar, 50 μm.

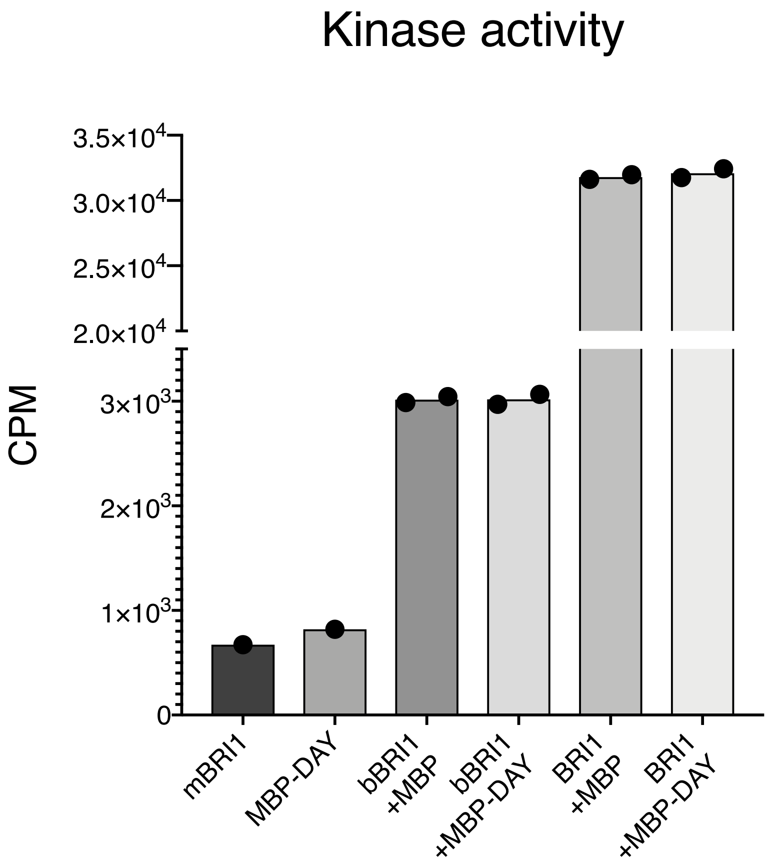

Supplementary Fig. 12 DAY does not enhance BRI1 kinase activity *in vitro.* *In vitro* kinase assays were performed with various forms of the cytosolic domain of BRI1 using the BR13 synthetic peptide as a substrate, in the presence or absence of MBP or MBP-DAY. Two technical replicates are shown for the kinase assays. mBRI1, the kinase dead form of BRI1 (K911E); bBRI1, basal state of BRI1 (Low phosphorylated BRI1;[3]); CPM, counts per minute.

3. Oh, M.-H., Clouse, S. D. & Huber, S. C. Tyrosine Phosphorylation of the BRI1 Receptor Kinase Occurs via a Post-Translational Modification and is Activated by the Juxtamembrane Domain. *Front. Plant Sci.* (2012). doi:10.3389/fpls.2012.00175

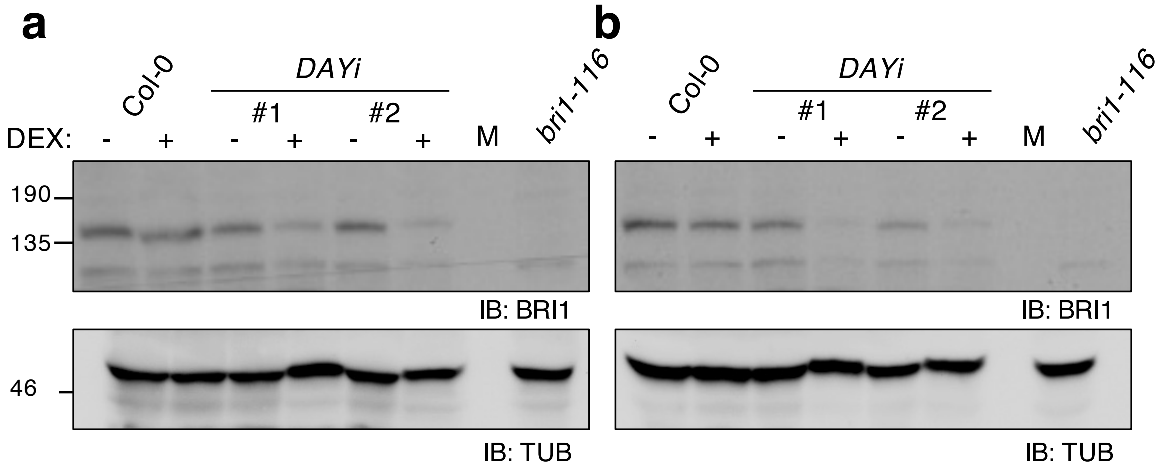

Supplementary Fig. 13 Biological replicate of Fig. 4b. Nine-day-old seedlings from WT (Col-0), *bri1* mutant (*bri1-116*), and *DAYi* plants grown in the presence of ethanol (-) or 1 µM DEX (+) were subjected to immunoblotting with anti-BRI1 and anti-α-tubulin (TUB) antibodies.

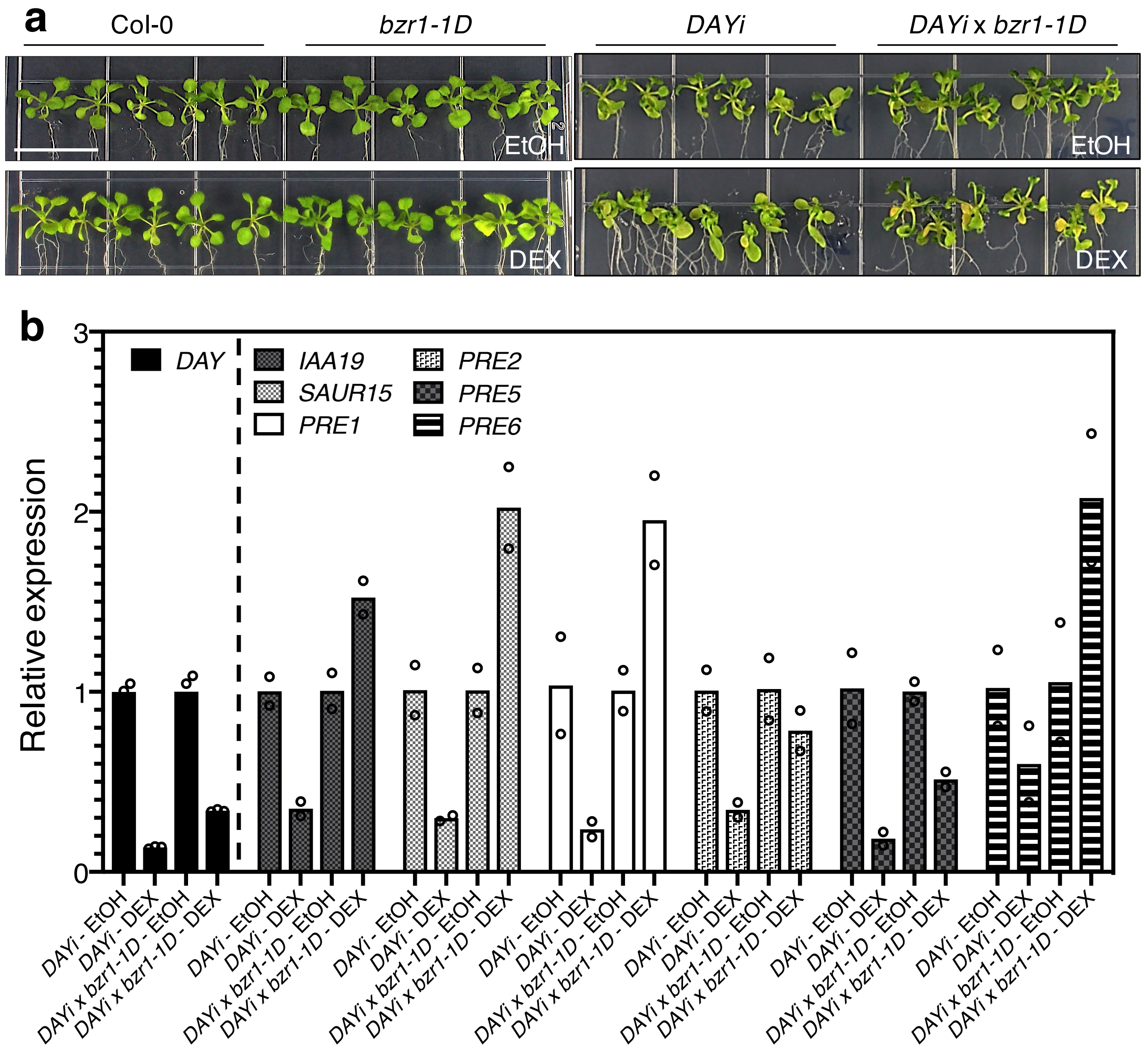

Supplementary Fig. 14 *bzr1-1D* rescues BR-deficient phenotypes in *DAY*-silenced seedlings. a, Seedlings from *DAYi* and *DAYi* x *bzr1-1D* lines were grown on ½ MS medium for 5 days at 22°C and then transferred to medium containing ethanol or 1 µM DEX at 28°C for 7 days. Scale bar for all pictures, 2 cm. **b**, RT-qPCR analysis of *DAY* and BR-related gene expression in *DAYi* and *DAYi* x *bzr1-1D* lines following ethanol or DEX treatment. Transcript levels were normalized to *PP2A A3* mRNA and expressed relative to EtOH-treated seedlings. (n = 2; technical replicates)

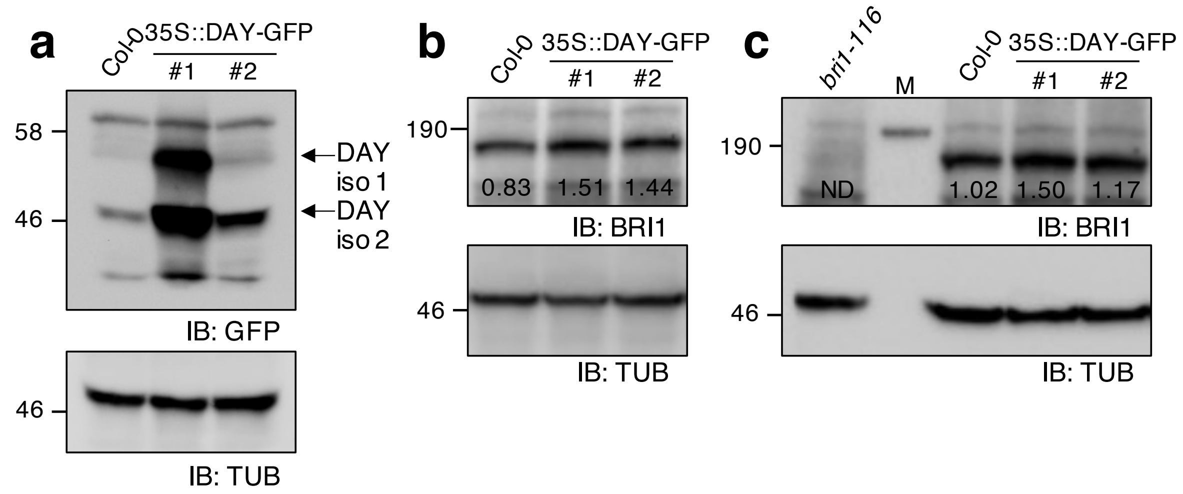

Supplementary Fig. 15 DAY enhances BRI1 accumulation in *Arabidopsis*. a, b, Overexpression of *DAY* increases the amount of BRI1 protein. Seven day old Col-0 and 35S::DAY-GFP seedlings grown on ½ MS medium were subjected to immunoblotting with anti-GFP (**a**) and anti-BRI1 (**b**) antibodies. For loading control, immunoblotting was performed with anti-α-tubulin (TUB) antibody. Numbers on the immunoblot of BRI1 indicate the relative amount of BRI1 compared to TUB. **c**, Biological replication of (**b**) with *bri1-116* mutation. ND; not detected.

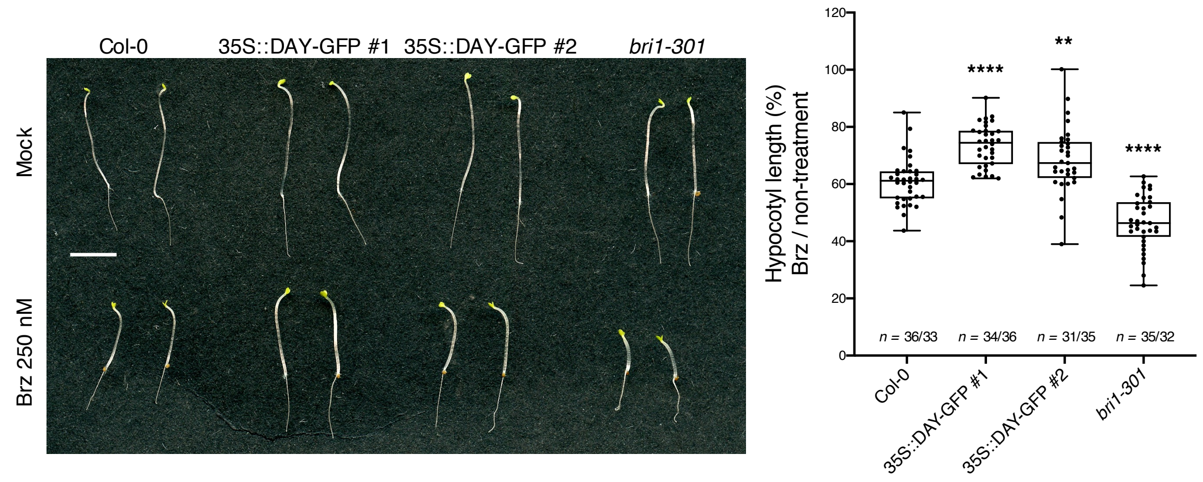

Supplementary Fig. 16 Hypocotyl elongation of *DAY* overexpression plants is less sensitive to BR biosynthesis inhibitor BRZ. *DAY* overexpression seedlings developed longer hypocotyls than those of WT upon Brz treatment. Seedlings were grown on medium with or without 250 nM Brz in the dark for 5 days. *n* denotes numbers of biologically independent hypocotyls, Brz- *(left number*) and mock-treated (*right number*). Statistical significance was determined by one-way ANOVA, Dunnett's multiple comparisons test **, $P \leq 0.01$; ****, $P < 0.0001$. Scale bar, 1 cm.

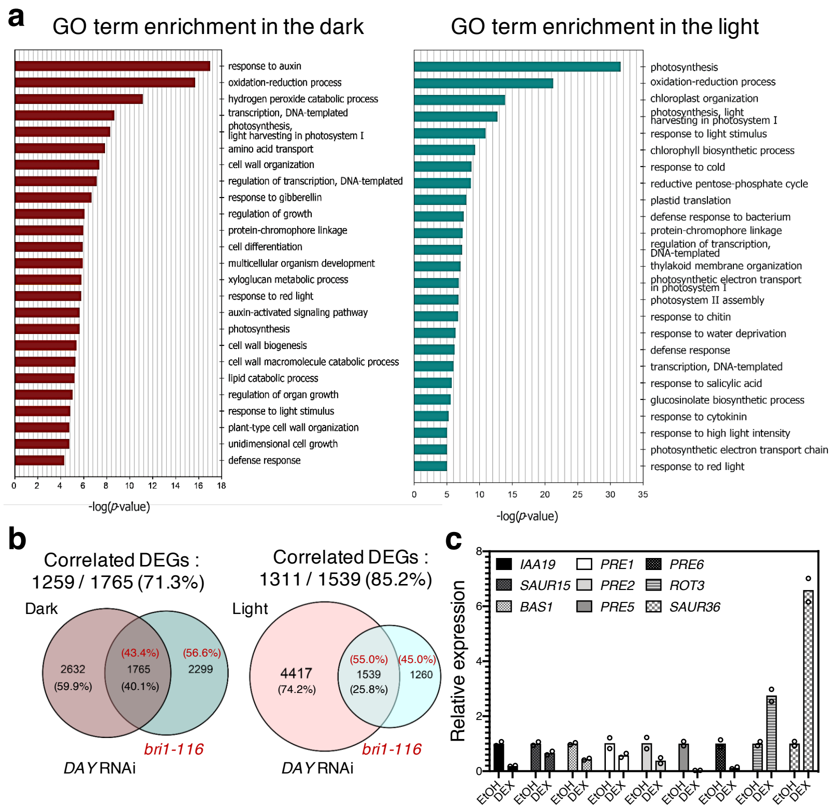

Supplementary Fig. 17 Transcriptome of *DAYi* seedlings significantly overlaps with the *bri1-116* mutant. a, Gene ontology (GO) term enrichment analyses for differentially expressed genes (DEGs) in *DAYi* seedlings in the dark (left panel) and in the light (right panel). In the dark, DAY-responsive genes primarily promoted growth, with genes classified in the response to the growth-promoting hormone auxin as the most significant; in the light, the most significant DAY-responsive genes were related to photosynthesis. In both the light and dark, the second most enriched group of DAY DEGs were those involved in oxidation-reduction processes, suggesting that DAY mainly regulates chloroplast development. During the transition of etioplasts to chloroplasts, most expressed proteins are involved in oxidation-reduction processes [4]. For each analysis, the top 25 enriched terms are presented based on their *p*-values from Fisher's exact test. The *x*-axis depicts the –log(*p*-value), for which a higher value represents more significant enrichment. **b**, Venn diagrams show a significant overlap between the *DAYi* and *bri1-116* transcriptomes in the dark and light. Of the 4397 DEGs in *DAYi* and 4064 in *bri1-116* identified in dark-grown seedlings, 1765 overlapped. Most were regulated in the same direction, showing either an increase or decrease in both mutants (correlated DEGs). In the light-grown seedlings, of the 5956 *DAYi* DEGs and 2799 *bri1-116* DEGs, 1539 were coregulated and 1311 of these were correlated. Numbers indicate DEGs. *Pink* and *light blue* circles represent the transcripts expressed in *DAYi* seedlings and the *bri1-116* mutant, respectively. Percentages in *black* and *red* colors (in parentheses) represent the % of DEGs from *DAYi* and *bri1-116*, respectively. **c**, To confirm the transcriptome data from *DAYi* plants, transcript levels of BR-activated and BR-repressed genes were determined by RT-qPCR analysis. *DAYi* seedlings were grown in the light for 9 days in the presence of ethanol or 1 μM DEX. (n = 2; technical replicates)

4. Kanervo, E. *et al.* Expression of protein complexes and individual proteins upon transition of etioplasts to chloroplasts in pea (Pisum sativum). *Plant Cell Physiol.* (2008). doi:10.1093/pcp/pcn016

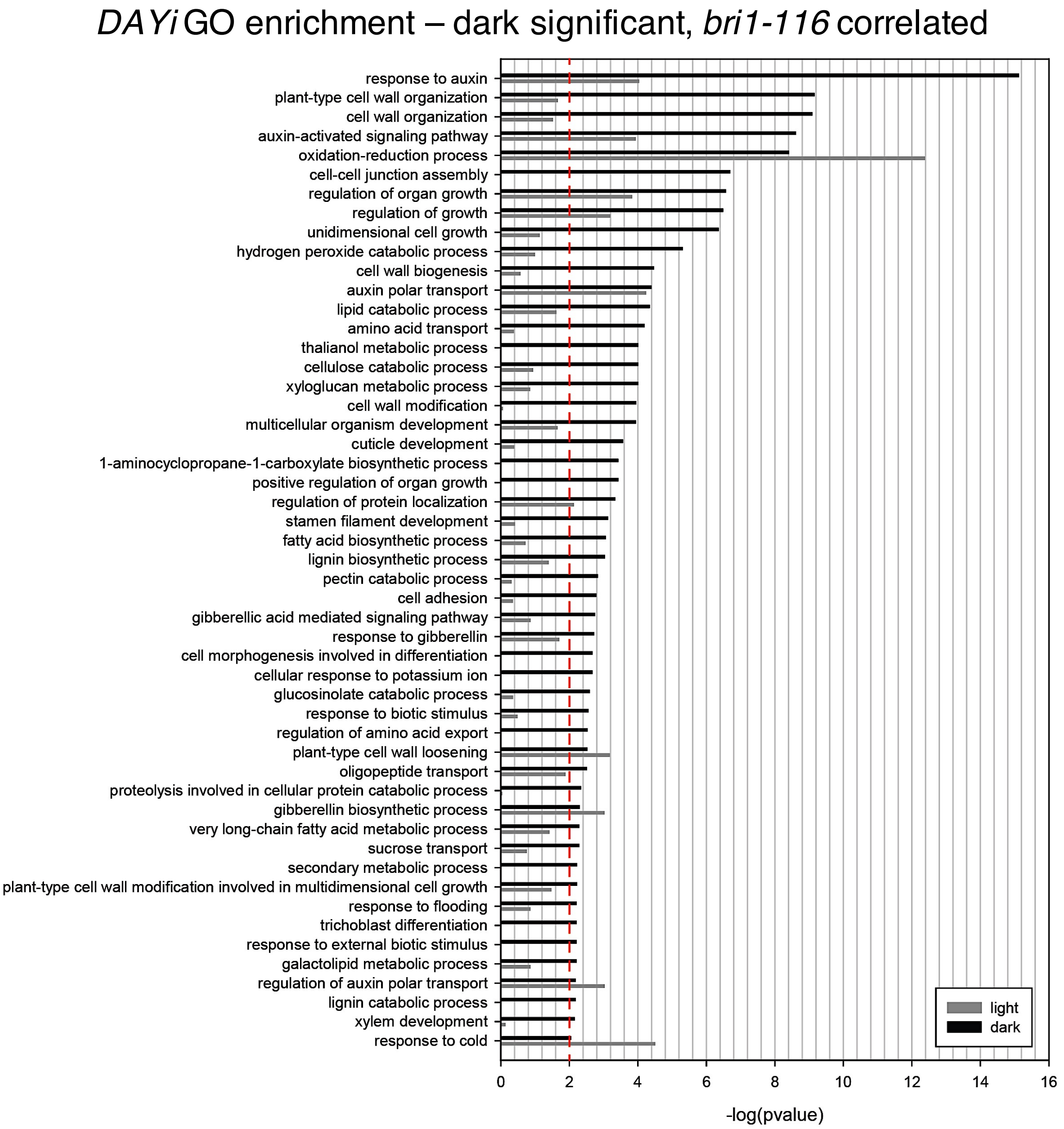

Supplementary Fig. 18 GO term enrichment analyses of *DAYi* DEGs in dark conditions. *DAYi* DEGs that were significantly correlated with those of *bri1-116* ($p \leq 0.01$; Fisher's exact test) in the dark are arranged in decreasing order based on their –log(p-value). *Black bars* represent enrichment in the dark, while *gray bars* represent enrichment in the light. *Red dotted-line* indicates the p-value threshold of 0.01. Correlated genes in dark conditions are enriched for GO terms related to plant growth promotion.

DAYi GO enrichment – light significant, *bri1-116* correlated

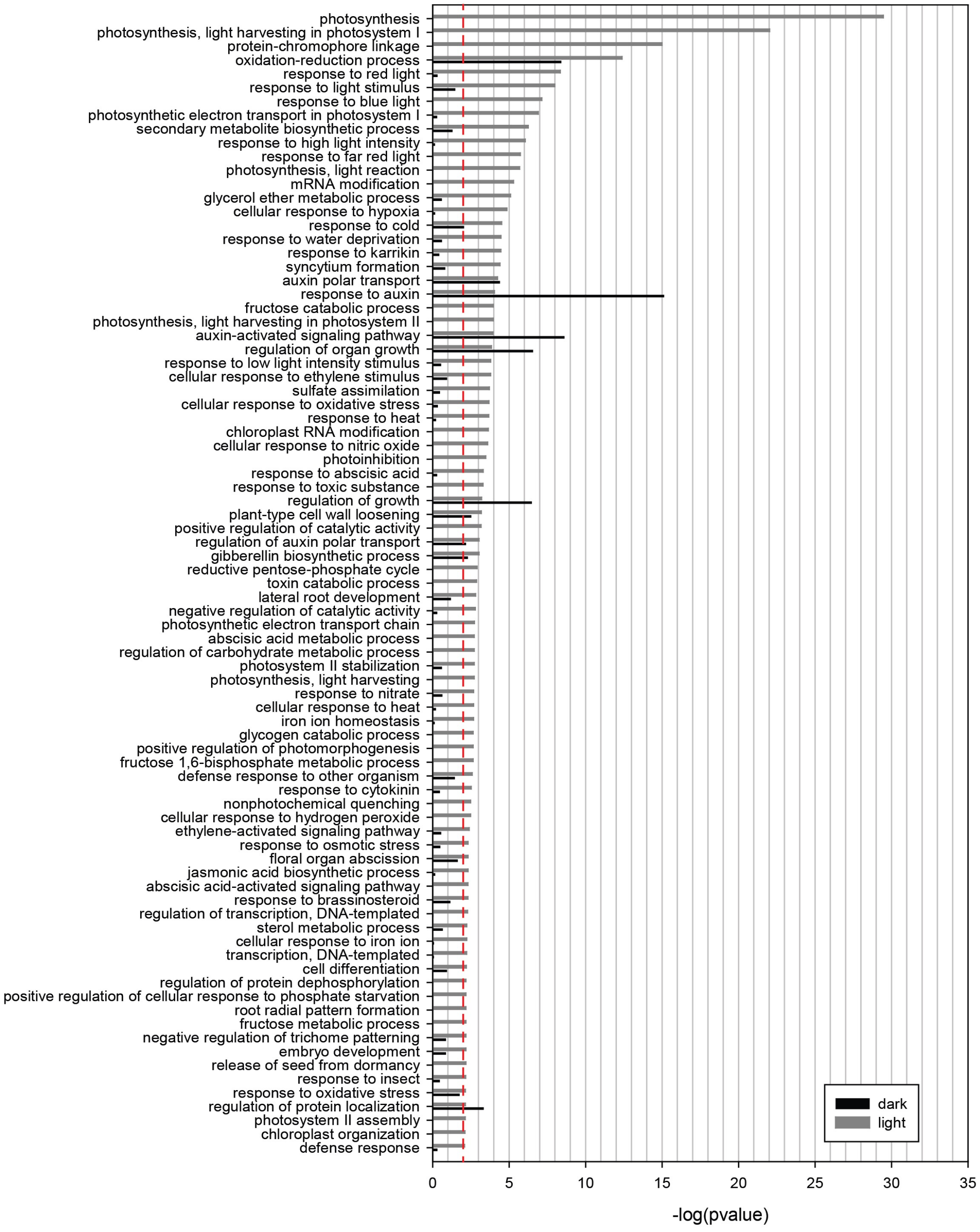

Supplementary Fig. 19 GO term enrichment analyses of *DAYi* DEGs in light conditions. *DAYi* DEGs that were significantly correlated with those of *bri1-116* ($p \leq 0.01$; Fisher's exact test) in the light are arranged in decreasing order based on their –log(p-value). *Black bars* represent enrichment in the dark, while *gray bars* represent enrichment in the light. *Red dotted-line* indicates the p-value threshold of 0.01. Correlated genes in light conditions are enriched for GO terms related to photosynthesis.

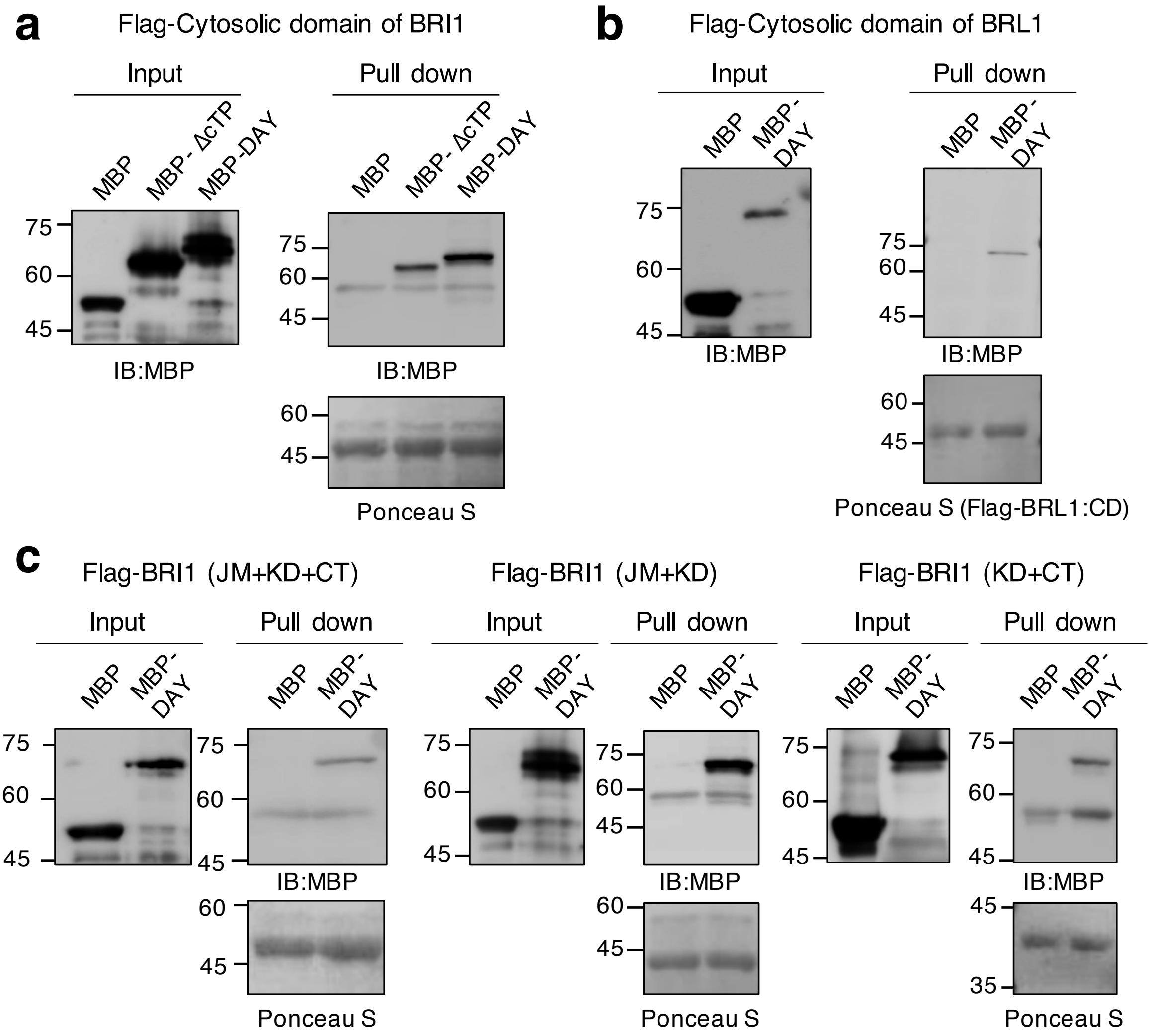

Supplementary Fig. 20 DAY directly interacts with the kinase domain of BRI1 *in vitro.* **a**, DAY interacts with the cytosolic domain of BRI1. MBP, MBP-DAY lacking the cTP (MBP-ΔcTP), and MBP-DAY were immunoprecipitated with the Flag-tagged cytosolic domain of BRI1 (Flag-BRI1:CD) immobilized on an anti-Flag affinity gel. Eluates were analyzed by immunoblotting using an anti-MBP antibody. Flag-BRI1:CD was visualized by Ponceau S staining. **b**, DAY binds to the cytosolic domain of BRI1-LIKE 1 (BRL1:CD). MBP and MBP-DAY were pulled down by Flag-BRL1:CD immobilized on anti-Flag affinity gel. Eluates were examined by immunoblotting using an anti-MBP antibody. Flag-BRL1:CD was visualized by Ponceau S staining. **c**, DAY interacts with the kinase domain of BRI1. MBP and MBP-DAY were pulled down by various forms of Flag-BRI1 proteins immobilized on anti-Flag affinity gel. Eluates were analyzed by immunoblotting using an anti-MBP antibody. Amounts of the different Flag-BRI1 proteins were visualized by Ponceau S staining. JM, juxta-membrane domain; KD, kinase domain; CT, C-terminal tail.
