# Supplementary material for: Chaperone-like protein DAY plays critical roles in photomorphogenesis
Source: Nat Commun. 2021 Jul 7;12:4194. doi: 10.1038/s41467-021-24446-5 (PMC8263706; doi:10.1038/s41467-021-24446-5)
Supplement: Supplementary file 2 — Descriptions of Additional Supplementary Files [file 41467_2021_24446_MOESM2_ESM.pdf]

Descriptions of Additional Supplementary Files

Supplementary Data 1
Description: List of plasmids

Supplementary Data 2
Description: List of primers

Supplementary Data 3
Description: Sequence of DAY in Nicotiana benthamiana
