# Supplementary material for: Chaperone-like protein DAY plays critical roles in photomorphogenesis
Source: Nat Commun. 2021 Jul 7;12:4194. doi: 10.1038/s41467-021-24446-5 (PMC8263706; doi:10.1038/s41467-021-24446-5)
Supplement: Supplementary file 6 — Reporting Summary [file 41467_2021_24446_MOESM6_ESM.pdf]

nature research

Corresponding author(s): Hyun-Sook Pai

Last updated by author(s): Jun 13, 2021

Reporting Summary

Nature Research wishes to improve the reproducibility of the work that we publish. This form provides structure for consistency and transparency in reporting. For further information on Nature Research policies, see our Editorial Policies and the Editorial Policy Checklist.

Statistics

For all statistical analyses, confirm that the following items are present in the figure legend, table legend, main text, or Methods section.

n/a	Confirmed	
☐	☒	The exact sample size (*n*) for each experimental group/condition, given as a discrete number and unit of measurement
☐	☒	A statement on whether measurements were taken from distinct samples or whether the same sample was measured repeatedly
☐	☒	The statistical test(s) used AND whether they are one- or two-sided<br>*Only common tests should be described solely by name; describe more complex techniques in the Methods section.*
☒	☐	A description of all covariates tested
☒	☐	A description of any assumptions or corrections, such as tests of normality and adjustment for multiple comparisons
☐	☒	A full description of the statistical parameters including central tendency (e.g. means) or other basic estimates (e.g. regression coefficient) AND variation (e.g. standard deviation) or associated estimates of uncertainty (e.g. confidence intervals)
☐	☒	For null hypothesis testing, the test statistic (e.g. F, t, r) with confidence intervals, effect sizes, degrees of freedom and P value noted<br>*Give P values as exact values whenever suitable.*
☒	☐	For Bayesian analysis, information on the choice of priors and Markov chain Monte Carlo settings
☒	☐	For hierarchical and complex designs, identification of the appropriate level for tests and full reporting of outcomes
☒	☐	Estimates of effect sizes (e.g. Cohen's d, Pearson's r), indicating how they were calculated

Our web collection on statistics for biologists contains articles on many of the points above.

Software and code

Policy information about availability of computer code

Data collection	Immunoblot images: ChemiDoc Touch Imaging system (v2.3.0.07), Bio-Rad<br>CLSM images: Zen (V 2.3), Carl Zeiss<br>Light scattering assay data:DU 800 system and applications (V.1.00) ; Beckman<br>RT-qPCR: LightCycler 96 (V 1.01.01.0050)
Data analysis	Microscopy images were processed in Zen (V 2.3) or Fiji (V 2.0)<br>Immunoblot quantifications were performed in Fiji (V 2.0)<br>Plants images quantifications were performed in Fiji (V 2.0)<br>CASAVA (V 1.8.2), Tophat (v2.0.13), and Cuffdiff (v2.2.0) software were used to analyze RNA-Seq data.<br>All other data/graphs were plotted and analysed using Excel or Prism 7.0

For manuscripts utilizing custom algorithms or software that are central to the research but not yet described in published literature, software must be made available to editors and reviewers. We strongly encourage code deposition in a community repository (e.g. GitHub). See the Nature Research guidelines for submitting code & software for further information.

Data

Policy information about availability of data

All manuscripts must include a data availability statement. This statement should provide the following information, where applicable:

- Accession codes, unique identifiers, or web links for publicly available datasets
- A list of figures that have associated raw data
- A description of any restrictions on data availability

RNA sequencing data were deposited into the Gene Expression Omnibus database under accession number GSE177028 and the NCBI Sequence Read Archive under

accession number SRP323708. The datasets generated during and/or analysed during the current study are available from the corresponding author on reasonable request.

Field-specific reporting

Please select the one below that is the best fit for your research. If you are not sure, read the appropriate sections before making your selection.

- [x] Life sciences
- [ ] Behavioural & social sciences
- [ ] Ecological, evolutionary & environmental sciences

For a reference copy of the document with all sections, see nature.com/documents/nr-reporting-summary-flat.pdf

Life sciences study design

All studies must disclose on these points even when the disclosure is negative.

Sample size	No statistical methods were used to predetermine sample sizes. Sample size was chosen as large as possible and in accordance with previous established protocols in the field.
Data exclusions	No data excluded
Replication	All experiment was repeated at least twice.
Randomization	Sampling of plants or leaf discs was performed by random selection or whole collection if possible.
Blinding	Blinding was not possible in this study. There were mostly microscopic images and biochemical assays.

Reporting for specific materials, systems and methods

We require information from authors about some types of materials, experimental systems and methods used in many studies. Here, indicate whether each material, system or method listed is relevant to your study. If you are not sure if a list item applies to your research, read the appropriate section before selecting a response.

Materials & experimental systems

n/a	Involved in the study	
[ ]	[x]	Antibodies
[x]	[ ]	Eukaryotic cell lines
[x]	[ ]	Palaeontology and archaeology
[x]	[ ]	Animals and other organisms
[x]	[ ]	Human research participants
[x]	[ ]	Clinical data
[x]	[ ]	Dual use research of concern

Methods

n/a	Involved in the study	
[x]	[ ]	ChIP-seq
[x]	[ ]	Flow cytometry
[x]	[ ]	MRI-based neuroimaging

Antibodies

Antibodies used	POR, cpHSP70, and BRI1 antibodies were purchased from Agrisera (AS05 067; 1:1000, AS08 348; 1:5000, and AS12 1859; 1:1000). Anti-α-Tubulin, GST, and MBP antibodies were purchased from Sigma-Aldrich (T6199; 1:5000, RPN1236; 1:5000, and M1321; 1:5000). GFP antibody was purchased from Miltenyi Biotec (130-091-833 1; 1:1000). Native DAY antibody (X-Q94C78-C; 1:500), was purchased from AB-mart (http://www.ab-mart.com). X-Q94C78-C mouse monoclonal antibody give raised with 3 amino acids from the C-terminal end of DAY protein sequence. Non-conjugated primary antibodies were detected using anti-mouse IgG-HRP (Sigma, A9044, 1:5000) or anti-rabbit IgG-HRP (Sigma, A6154, 1:10000).
Validation	Validation statements of commercial primary antibodies are available from each manufacturers.<br>POR (https://www.agrisera.com/en/artiklar/por-protochlorophilide-oxidoreductase.html)<br>cpHSP70 (https://www.agrisera.com/en/artiklar/hsp70-heat-shock-protein-70-chloroplastic-.html)<br>BRI1 (https://www.agrisera.com/en/artiklar/bri1-brassinosteroid-insensitive-1.html)<br>Flag (https://www.sigmaaldrich.com/catalog/product/sigma/a8592?lang=de&region=AT)<br>α-Tubulin (https://www.sigmaaldrich.com/catalog/product/sigma/t6199?lang=de&region=AT)<br>GST (https://www.sigmaaldrich.com/catalog/product/sigma/gerpn1236?lang=de&region=AT)<br>MBP (https://www.sigmaaldrich.com/catalog/product/sigma/m1321?lang=de&region=AT)<br>GFP (https://www.miltenyibiotec.com/AT-en/products/gfp-antibody-gg4-2c2-12-10.html#gref)<br>DAY native antibody were validated in figure 1b.
